# Seroprevalence of Q fever among humans and animals in East Africa: a systematic review and meta-analysis

**DOI:** 10.1186/s42522-026-00209-4

**Published:** 2026-04-03

**Authors:** Simegnew Adugna Kallu, Ambachew Motbaynor Wubaye, Simachew Getaneh Endalamew, Andnet Yirga Assefa, Alebachew Tilahun Wassie, Migbaru Keffale Bezabih, Yihenew Getahun Ambaw

**Affiliations:** 1 College of Veterinary Medicine, Haramaya University, P. O. Box 138, Dire Dawa, Ethiopia; 2 Department of Veterinary Science, College of Agriculture and Environmental Science, Debre Tabor University, P.O. Box 272, Debre Tabor, Ethiopia; 3 Department of Veterinary Epidemiology and Public Health, School of Veterinary Medicine, Bahir Dar University, Bahir Dar, Ethiopia; 4 Department of Veterinary Clinical Medicine, School of Veterinary Medicine, Bahir Dar University, Bahir Dar, Ethiopia; 5 Armauer Hansen Research Institute, Addis Ababa, Ethiopia; 6 Department of Veterinary Medicine, College of Agricultural Sciences, Woldia University, Weldiya, Ethiopia

**Keywords:** *C. burnetii*, Q fever, Seroprevalence, One health, Meta-analysis, Human, Animals, East Africa

## Abstract

Q fever is a neglected zoonotic disease caused by *Coxiella burnetii* with a global distribution. It has a significant public health implication in resource-limited settings. Therefore, this systematic review and meta-analysis were designed to comprehensively analyze the seroprevalence of Q fever in humans and animals in East Africa. Following the PRISMA guidelines and a registered protocol on PROSPERO (CRD420251149865), online searches were conducted in Pubmed, ScienceDirect, Scopus, Web of Science, Google Scholar, and AJOL from October 1–5, 2025, to retrieve articles from the East African countries published within the time frame of January 1, 2000 and September 30, 2025. This systematic review and meta-analysis identified 47 articles reporting Q fever seroprevalence across multiple animal species and human, yielding 94 species-specific datasets for pooled analysis. After heterogeneity assessment, random effects model was used to estimate the pooled Q fever seroprevalence. The estimated pooled seroprevalence was 21.32% (95% CI: [14.58; 28.06]) among humans and 27.55% (95% CI: [22.34; 32.77]) in animals. Trim-and-fill analysis revealed substantial publication bias, reducing pooled seroprevalence from 27.55% to 8.46% in animals and from 21.35% to 6.98% in humans. The seroprevalence in humans was higher in Kenya (26.0%) and Ethiopia (24.7%) than in Sudan (18.0%), Tanzania (14.9%), and Uganda (7.6%). The multivariable meta-regression revealed a significant increasing trend in human Q fever seroprevalence over time, despite marginal non-significance in univariable analysis. Among animals, the seroprevalence was higher in camels (38.9%) and lower in cattle (14.6%), with observed statistically significant difference. This meta-analysis result showed that the seroprevalence of Q fever is high in humans and animals, but it is still neglected in many countries in the East Africa, posing significant impact to human and animal health. Planned preventive measures and control efforts are necessary.

**Supplementary Information:**

The online version contains supplementary material available at 10.1186/s42522-026-00209-4.

## Introduction

Query (Q) fever is a neglected zoonotic disease caused by an intracellular, Gram-negative pathogenic bacterium *Coxiella burnetii* [1]. The disease has worldwide distribution and *C. burnetii* is a category B bioterrorism agent with a wide and diverse host range including human, domestic livestock and pets, wildlife, reptiles, fish, birds, and ticks [2, 3]. Domestic ruminants including cattle, goats, sheep, and camel are the main reservoirs and sources of human infections [4–8].

Although Q fever in animals often remain subclinical, it leads to abortion, stillbirth, premature birth, and infertility [9], which ultimately result in decreased productivity and cause substantial economic loss for farmers, particularly in low-resource settings [10–13]. In addition, the persistent presence of *C. burnetii* in animal populations sustains zoonotic risk to humans through close contact and environmental exposure.

Q fever is primarily transmitted to humans and animals through the inhalation of aerosols contaminated with *C. burnetii*, which are generated from birth products, urine, feces, and dust contaminated by infected animals [14, 15]. During parturition, large amounts of bacteria are shed in amniotic fluid and fetal membranes, facilitating environmental contamination and subsequent aerosol transmission [2, 16]. In addition to inhalation of aerosols, humans can acquire the infection through ingestion of unpasteurized milk and handling contaminated materials like straw, manure, or farm equipment [16]. Despite *C. burnetii* infecting all social groups, Q fever in humans predominantly affects individuals who interact with livestock, including farmers, herders, slaughterhouse workers, and veterinarians, and is considered an occupational disease [17].

The East Africa region contains more than 50% of Africa’s livestock population [18]. Cattle, sheep, goats, and camels are among the most important livestock species used for meat and milk production and are considered as the critical component of East Africa’s economy. However, the unique ecological and socio-economic conditions of the East African region present conducive situations to *C. burnetii* transmission. Extensive pastoral and agro-pastoral livestock production systems, large-scale animal movements across borders during droughts and for trade purposes, and close human-animal contact during routine husbandry and market activities all amplify the risk of zoonotic spillover [19]. Traditional practices, including manual assistance during animal births, handling of placental tissues and abortion materials without protective equipment, and consumption of unpasteurized milk, substantially increase occupational and household exposure risk [20]. Moreover, environmental factors, including aridity, seasonal rainfall patterns, and wind persistence, affect *C. burnetii* environmental survival and aerosolization capacity, facilitating pathogen dispersal across considerable distances [21].

Despite global recognition of Q fever as a notable zoonosis, its burden and epidemiological distribution in developing regions, particularly in Africa, remain poorly characterized [22]. Studies from Europe, North America, and Australia have well-documented the disease’s epidemiology, demonstrating major outbreaks and high seroprevalence in both animals and humans [19, 23, 24]. However, in East Africa, reporting and surveillance systems are inadequate, laboratory diagnostic capabilities remain limited, and the disease is often overshadowed by other febrile illnesses such as malaria, typhoid, or brucellosis [25].

Q fever diagnosis primarily relies on serological tests such as indirect immunofluorescence assay (IFA), complement fixation test (CFT), and enzyme-linked immunosorbent assay (ELISA), which detect antibodies against *C. burnetii*. Most commercial serological diagnostic tests for Q fever detect both phase I and phase II antibodies against *C. burnetii* simultaneously [26]. ELISA, the most widely used method, measures total IgG/IgM against phase II antigens and indicates past exposure but cannot distinguish acute, chronic, or historical seropositivity. Indirect immunofluorescence assay (IFA) is the gold standard, providing phase-specific titration, though subjective interpretation limits reliability. PCR-based assays are also used to detect *C. burnetii* DNA in clinical samples [14].

Although sporadic seroepidemiological surveys and outbreak investigations have been conducted in some East African countries, findings remain fragmented and inconsistent. Reported prevalence estimates vary widely across species, regions, and diagnostic methods used [22]. These gaps hinder the ability of policymakers and health authorities to develop evidence-based control and prevention strategies aligned with the One Health framework in East Africa.

Coordinated surveillance, improved diagnostic capacity, and joint response frameworks can facilitate early detection and reduce Q fever transmission at the human-animal interface [11, 27–31]. However, such interventions necessitate baseline data regarding the distribution and prevalence of infection across various host species and countries. Currently, available data remain inadequate and scattered among individual studies, with no previous comprehensive meta-analysis has examining the seroprevalence of Q fever in humans and animals across East Africa. By consolidating findings from diverse studies, this systematic review and meta-analysis aims to provide a comprehensive regional estimate of Q fever seroprevalence in East Africa. This information is intended to guide veterinary and public health strategies for the effective management of the disease and support the implementation of integrated One Health interventions targeting both human and animal populations.

## Methods

### Study design

The Preferred Reporting Items for Systematic Reviews and Meta-Analyses (PRISMA) 2020 checklist was used to identify, select, appraise, and synthesize studies and for reporting evidence-based systematic review and meta-analysis [32]. To ensure comprehensive and focused literature inclusion and data synthesis, the condition, context, and population (CoCoPo) outline was used [33]. To ensure transparency, reduce bias, and avoid unintended duplication, the protocol for this systematic review and meta-analysis was registered in the International Prospective Register of Systematic Reviews (PROSPERO; registration number: CRD420251149865) before starting the review process.

### Literature search strategy

Studies conducted about the seroprevalence of Q fever in humans and animals were searched in major databases (PubMed, Scopus, Web of Science, Science Direct, AJOL, Google Scholar) using advanced search options. The search was conducted using the keywords ((“Q fever” OR “*Coxiella burnetii*” OR “*C. burnetii*” OR “Coxiellosis”) AND (“prevalence” OR “seroprevalence” OR “serosurvey” OR “epidemiology” OR “infection”) AND (“humans” OR “man” OR “people” OR “patients” OR “livestock” OR “animals” OR “ruminants” OR “cattle” OR “bovine” OR “goats” OR “caprine” OR “sheep” OR “ovine” OR “camels” OR “dromedary”) AND (“East Africa” OR “Ethiopia” OR “Kenya” OR “Uganda” OR “Tanzania” OR “Somalia” OR “Sudan” OR “South Sudan” OR “Burundi” OR “Eritrea” OR “Djibouti” OR “Comoros” OR “Rwanda” OR “Seychelles”)). All relevant published articles on Q fever in humans and animals in East African countries between 1 January 2000 and 30 September 2025 were retrieved, and the search was done from October 1–5, 2025.

### Eligibility criteria, selection process, and data extraction

Eligible published articles were selected based on a predefined criteria including studies conducted in East Africa region (according to the African Development Bank Group East Africa includes countries: Ethiopia, Kenya, Uganda, Tanzania, South Sudan, Sudan, Somalia, Burundi, Eritrea, Djibouti, Comoros, Rwanda, and Seychelles) (Supplementary Fig. S1), published between 1 January 2000 and 30 September 2025, specified total number of cattle, sheep, goats, camels, and humans sampled, total number of animals and humans tested positive for Q fever.

**Fig. 1:**
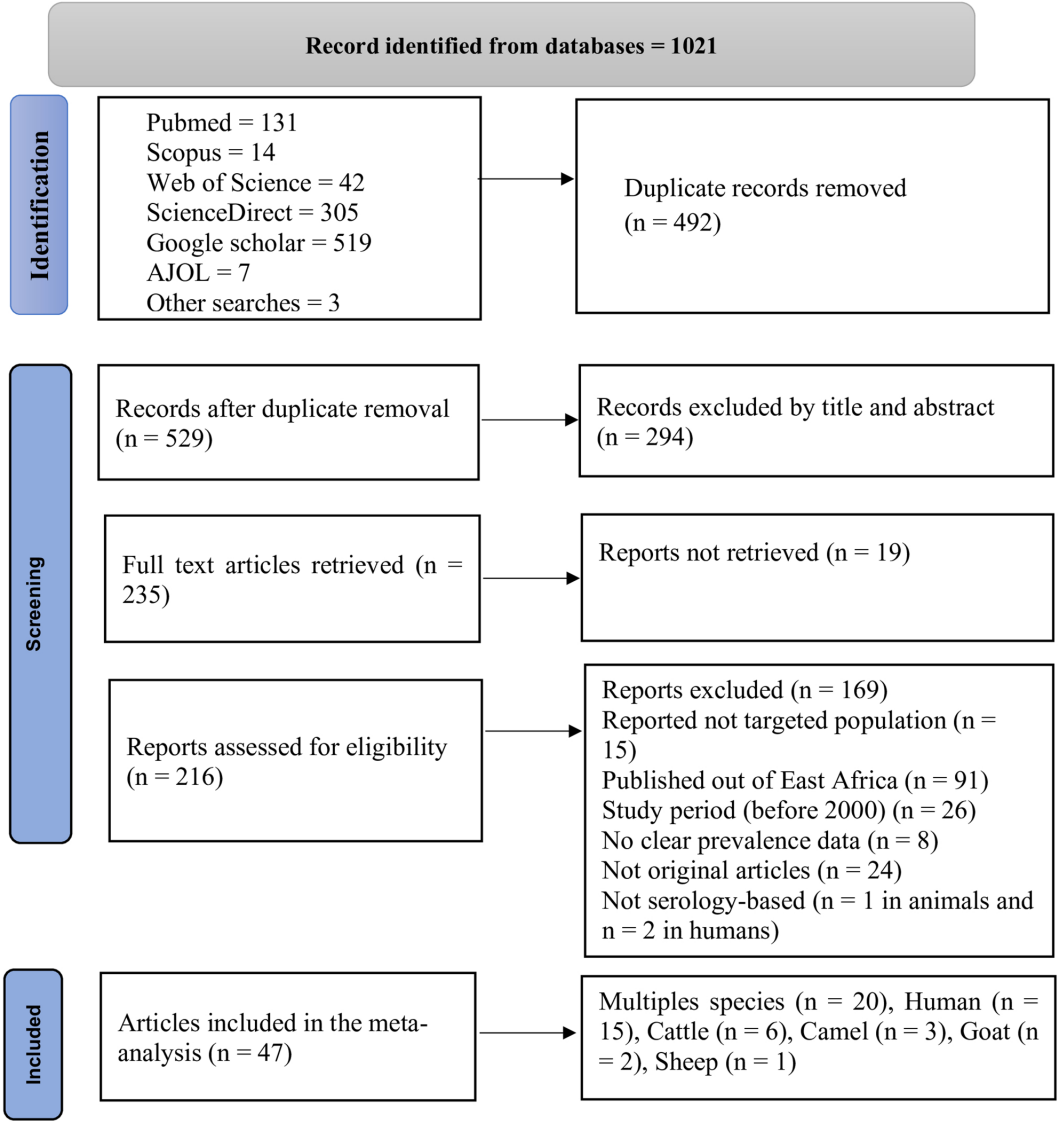
PRISMA flow diagram of the selection of eligible studies

The selection process was conducted based on the PRISMA flowchart, showing the total number of studies retrieved, those included in this study, and excluded from analysis with the reason of exclusion (Fig. 1). Two independent teams (MKB and YGA; ATW and AYA) screened the studies based on their title and abstract and further assessed the full text of the studies based on the above criteria to decide on the inclusion and exclusion of the study. Disagreements in the inclusion and exclusion of articles were resolved through discussions.

After excluding articles that did not meet the inclusion criteria, 47 articles were selected for the analysis. Two authors (SAK and SGE) independently extracted data using a standardized Excel format including title, authors, year of publication, country of study, species, detection method, number of animals and humans tested, and number of animals and humans tested positive. When an article published data from more than one target population, the data was extracted for each target population and the article divided according to the number of targeted populations.

### Quality assessment

The quality of eligible publications included in this systematic review and meta-analysis was evaluated by two reviewers (SAK and AMW) using the Joanna Briggs Institute (JBI) critical appraisal tool for the prevalence studies [33]. This tool consists of nine items that assess the internal validity and potential sources of bias and each item was scored as “Yes”, “No”, “Unclear”, or “Not Applicable” (Supplementary Tables S1 and S2). Any disagreements between the two reviewers were resolved through discussion or consultation with a third reviewer. Quality scores were derived by assigning 1 point to each “Yes” or “Not Applicable” response and 0 points to “No”, “Unclear” or missing information across all nine JBI items. Then, studies were classified according to their total score distribution as high quality (7–9 points), moderate quality (4–6 points), and low quality (0–3 points) (Tables 1 and 2). Since all included studies in our systematic review and meta-analysis achieved moderate or high quality scores (ranging from 4 to 9 points), no studies were excluded based on inadequate methodology, and the overall body of evidence was considered sufficiently robust for synthesis.

### Statistical analysis

All the analysis was performed using R programming language version 4.5.1 [34]. The pooled seroprevalence of Q fever was determined using the “Meta” package. Heterogeneity between studies was assessed using Cochrane Q statistics, I^2^ [35], τ², and prediction intervals [36]. The random effects model was used when high heterogeneity is detected. Subgroup analysis was applied to explore the source of heterogeneity that contributes to the variation between studies. The potential sources of heterogeneity in human and animal studies were country, detection method, study period, and sample size. In addition to these, species was a potential source of heterogeneity in Q fever seroprevalence studies in animals.

To determine publication bias, a graph visualizing symmetry of the funnel plot and statistical tests of Egger’s weighted regression [37] and Begg’s rank correlation statistics [38] were used. Sensitivity analysis was performed to check the robustness and stability of the meta-analysis. Baujat plot was used to visually identify individual studies that contribute most to the overall heterogeneity and pooled effect estimate in the meta-analysis [39]. A leave-one-out meta-analysis was conducted as a sensitivity analysis by sequentially excluding each study and recalculating the pooled effect size and heterogeneity statistics [40]. This approach quantified how omitting each study influenced the pooled estimates and heterogeneity, thereby evaluating the robustness of the meta-analysis and identifying potentially influential or outlier studies.

A univariable mixed effects meta-regression analysis was performed on moderators to investigate potential sources of heterogeneity between the studies and to identify whether specific study features could explain the observed variability of seroprevalence estimates. Each study level characteristics, including country, detection method, sample size, study period, and species of the animal, was examined to assess its association with the effect sizes using a random-effects meta-regression model. Predictor variables with a p-value less than 0.25 were included in the multivariable meta-regression analysis. The presence of multicollinearity between the predictors was assessed using variance inflation factors (VIF) in “vif” function of the “performance” package and Goodman and Kruskal’s gamma statistic in the “GoodmanKruskalgamma” function of “DescTools” package. Absence of multicollinearity between predictors was considered when the VIF is less than 5 [41]. For categorical predictors, Goodman and Kruskal’s gamma statistic was additionally used, and gamma values between − 0.6 and 0.6 were considered as non-collinear predictors [42].

## Result

### Literature search results and quality assessment of the included studies

Based on the inclusion and exclusion criteria, 47 articles were found eligible. These studies conducted in six East African countries, assessed Q fever seroprevalence in humans, cattle, camels, sheep, and goats. After extracting species-specific data from each article, the number of studies increased to 94, since many articles reported results for multiple species.

The distribution of studies of Q fever in animals by country were 37 (51.4%), 25 (34.7%), 4 (5.6%), 3 (4.2%), and 3 (4.2%) from Kenya, Ethiopia, Tanzania, Sudan, and Somalia, respectively (Table 1). For humans, 22 studies were included in the analysis, ten were from Kenya, five from Tanzania, three from Sudan, three from Ethiopia, and one from Uganda (Table 2).

**Table 1:** Studies included in the analysis of Q fever seroprevalence in animals

Authors	Animal species	*N* tested	*N* positive	Prevalence (95% CI)	Country	Detection method	Quality score
Milkesa et al., 2024 [43]	Cattle	1564	185	11.8 (10.29, 13.56)	Ethiopia	ELISA	High
	Camel	736	209	28.4 (25.19, 31.83)	Ethiopia	ELISA	
	Sheep	828	167	20.2 (17.52, 23.10)	Ethiopia	ELISA	
	Goat	1012	216	21.3 (18.88, 24.03)	Ethiopia	ELISA	
Alamerew et al., 2022 [44]	Goat	35	23	65.7 (47.74, 80.32)	Ethiopia	ELISA	Moderate
Deressa et al., 2020 [45]	Cattle	422	37	8.8 (6.33, 11.99)	Ethiopia	ELISA	High
Gebretensay et al., 2019 [46]	Sheep	445	169	38.0 (33.48, 42.69)	Ethiopia	ELISA	High
Tesfaye et al., 2020 [47]	Goat	293	104	35.5 (30.07, 41.30)	Ethiopia	ELISA	High
	Sheep	213	39	18.3 (13.49, 24.30)	Ethiopia	ELISA	
Oakley et al., 2024 [30]	Goat	684	360	52.6 (48.81, 56.42)	Ethiopia	IFA	High
	Sheep	199	85	42.7 (35.80, 49.91)	Ethiopia	IFA	
	Cattle	236	23	9.7 (06.41, 14.44)	Ethiopia	IFA	
	Camel	258	42	16.3 (12.11, 21.48)	Ethiopia	IFA	
Girmay et al., 2024 [48]	Cattle	164	1	0.6 (0.03, 3.86)	Ethiopia	ELISA	High
Gumi et al., 2013 [49]	Cattle	180	57	31.7 (25.06, 39.07)	Ethiopia	ELISA	Moderate
	Camel	90	81	90.0 (81.41, 95.04)	Ethiopia	ELISA	
	Goat	98	53	54.1 (43.75, 64.09)	Ethiopia	ELISA	
Ibrahim et al., 2021 [50]	Cattle	108	11	10.2 (5.44, 17.87)	Ethiopia	ELISA	High
	Sheep	229	69	30.1 (24.35, 36.59)	Ethiopia	ELISA	
	Goat	252	123	48.8 (42.51, 55.15)	Ethiopia	ELISA	
	Camel	141	79	56.0 (47.43, 64.29)	Ethiopia	ELISA	
Getachew et al., 2024 [51]	Cattle	450	169	37.6 (33.10, 42.23)	Ethiopia	ELISA	High
	Goat	450	165	36.7 (32.24, 41.33)	Ethiopia	ELISA	
	Sheep	450	93	20.7 (17.08, 24.77)	Ethiopia	ELISA	
Robi et al., 2024 [52]	Cattle	461	40	8.7 (6.34, 11.72)	Ethiopia	ELISA	High
Nakeel et al., 2016 [53]	Cattle	156	140	89.7 (83.61, 93.84)	Kenya	ELISA	Moderate
	Sheep	80	46	57.5 (45.95, 68.32)	Kenya	ELISA	
	Goat	83	69	83.1 (72.98, 90.15)	Kenya	ELISA	
Knobel et al., 2013 [54]	Cattle	463	131	28.3 (24.28, 32.67)	Kenya	IFA	High
	Goat	378	121	32.0 (27.38, 37.01)	Kenya	IFA	
	Sheep	159	29	18.2 (12.74, 25.31)	Kenya	IFA	
Rooney et al., 2024 [55]	Camel	233	121	51.9 (45.32, 58.47)	Kenya	ELISA	High
Muema et al., 2022 [56]	Goat	1876	1402	74.7 (72.69, 76.67)	Kenya	ELISA	
	Sheep	322	183	56.8 (51.22, 62.28)	Kenya	ELISA	High
	Camel	189	73	38.6 (31.72, 45.99)	Kenya	ELISA	
Mutisya, 2024 [57]	Cattle	258	18	7.0 (4.31, 10.99)	Kenya	ELISA	High
	Camel	97	28	28.9 (20.34, 39.09)	Kenya	ELISA	
	Goat	953	635	66.6 (63.52, 69.60)	Kenya	ELISA	
	Sheep	849	352	41.5 (38.13, 44.86)	Kenya	ELISA	
Wambua et al., 2025 [58]	Cattle	6593	521	7.9 (7.27, 8.59)	Kenya	ELISA	High
DePuy et al., 2014 [59]	Cattle	113	3	2.7 (0.69, 8.14)	Kenya	ELISA	Moderate
	Sheep	23	4	17.4 (5.72, 39.55)	Kenya	ELISA	
	Goat	26	9	34.6 (17.94, 55.64)	Kenya	ELISA	
	Camel	72	25	34.7 (24.14, 46.94)	Kenya	ELISA	
Mwololo et al., 2022 [60]	Cattle	466	14	3.0 (1.72, 5.11)	Kenya	ELISA	High
	Goat	1333	203	15.2 (13.36, 17.30)	Kenya	ELISA	
	Sheep	928	132	14.2 (12.07, 16.68)	Kenya	ELISA	
Muturi et al., 2021 [61]	Camel	120	25	20.8 (14.18, 29.40)	Kenya	ELISA	High
Muema et al., 2017 [62]	Goat	508	132	26.0 (22.27, 30.07)	Kenya	ELISA	High
	Sheep	332	40	12.0 (8.84, 16.16)	Kenya	ELISA	
Watene, 2021 [63]	Cattle	589	11	1.9 (0.99, 3.42)	Kenya	ELISA	High
Kiptanui et al., 2022 [64]	Cattle	725	59	8.1 (6.30, 10.43)	Kenya	ELISA	High
	Sheep	283	4	1.4 (0.45, 3.82)	Kenya	ELISA	
	Goat	132	1	0.8 (0.04, 4.77)	Kenya	ELISA	
Browne et al., 2017 [65]	Camel	334	62	18.6 (14.62, 23.24)	Kenya	ELISA	High
Larson et al., 2019 [66]	Cattle	157	9	5.7 (2.82, 10.93)	Kenya	ELISA	High
	Camel	312	62	19.9 (15.68, 24.83)	Kenya	ELISA	
	Goat	280	51	18.2 (13.97, 23.35)	Kenya	ELISA	
	Sheep	100	13	13.0 (7.38, 21.56)	Kenya	ELISA	
Wardrop et al., 2016 [67]	Cattle	955	100	10.5 (8.64, 12.63)	Kenya	ELISA	High
Osman et al., 2025 [68]	Cattle	133	0	0.0 (0.0, 3.50)	Somalia	IFA	Moderate
	Goat	190	40	21.1 (15.63, 27.68)	Somalia	IFA	
	Sheep	49	7	14.3 (6.41, 27.68)	Somalia	IFA	
Wainaina et al., 2022 [69]	Goat	228	58	25.4 (20.02, 31.70)	Kenya	ELISA	High
	Sheep	88	8	9.1 (4.29, 17.62)	Kenya	ELISA	
Hussien et al., 2017 [70]	Cattle	244	73	29.9 (24.33, 36.15)	Sudan	ELISA	Moderate
	Camel	76	49	64.5 (52.59, 74.88)	Sudan	ELISA	
Hussien et al., 2012 [71]	Goat	460	109	23.7 (19.94, 27.90)	Sudan	ELISA	Moderate
Bwatota et al., 2023 [72]	Cattle	2049	79	3.9 (3.08, 4.81)	Tanzania	ELISA	Moderate
Thomas et al., 2022 [73]	Cattle	3602	187	5.2 (4.50, 5.98)	Tanzania	ELISA	High
	Goat	3325	928	27.9 (26.40, 29.47)	Tanzania	ELISA	
	Sheep	2620	456	17.4 (15.98, 18.92)	Tanzania	ELISA	

N: number; CI: confidence interval

**Table 2:** Studies included in the analysis of Q fever prevalence in humans

Authors	Number tested	Number positive	Prevalence (95% CI)	Country	Detection method	Quality score
Oakley et al., 2024 [30]	335	91	27.2 [22.47; 32.27]	Ethiopia	IFA	High
Ibrahim et al., 2021 [50]	188	50	26.6 [20.43; 33.52]	Ethiopia	ELISA	High
Marami et al., 2025 [74]	402	86	21.4 [17.48; 25.73]	Ethiopia	ELISA	High
Nakeel et al., 2016 [53]	90	24	26.7 [17.89; 37.03]	Kenya	ELISA	Moderate
Knobel et al., 2013 [54]	246	76	30.9 [25.18; 37.08]	Kenya	IFA	High
Mutisya, 2024 [57]	683	305	44.7 [40.88; 48.47]	Kenya	ELISA	High
Cook et al., 2021 [75]	566	210	37.1 [33.11; 41.23]	Kenya	ELISA	High
Njeru et al., 2016 [76]	1067	194	18.2 [15.91; 20.63]	Kenya	ELISA	High
Mwololo et al., 2022 [60]	974	238	24.4 [21.77; 27.26]	Kenya	ELISA	High
Lemtudo et al., 2021 [77]	405	73	18.0 [14.40; 22.12]	Kenya	ELISA	Moderate
Wardrop et al., 2016 [67]	2049	52	2.5 [1.90; 3.31]	Kenya	ELISA	High
Wainaina, et al., 2024 [78]	216	99	45.8 [39.06; 52.73]	Kenya	ELISA	High
Maina et al., 2016 [79]	364	47	12.9 [9.64; 16.80]	Kenya	ELISA	Moderate
Boodman et al., 2025a [80]	560	29	5.2 [3.50; 7.35]	Sudan	IFA	High
Abbas et al., 2024 [81]	90	37	41.1 [31.84; 51.98]	Sudan	ELISA	Moderate
Boodman et al., 2025b [82]	333	31	9.3 [6.41; 12.95]	Sudan	IFA	Moderate
Crump et al., 2013 [83]	482	24	5.0 [3.22; 7.32]	Tanzania	ELISA	Moderate
Pisharody et al., 2022 [84]	344	16	4.7 [2.68; 7.44]	Tanzania	IFA	High
Moorthy et al., 2024 [85]	228	117	51.3 [44.63; 57.97]	Tanzania	IFA	Moderate
Prabhu et al., 2011 [86]	483	24	5.0 [3.21; 7.30]	Tanzania	ELISA	Moderate
Fiorillo et al., 2013 [87]	142	13	9.2 [4.97; 15.15]	Tanzania	ELISA	Moderate
Eneku et al., 2023 [88]	460	35	7.6 [5.36; 10.42]	Uganda	ELISA	High

CI: confidence interval

### Pooled seroprevalence and subgroup meta-analysis of Q fever in animals

The pooled seroprevalence of Q fever in this systematic review and meta-analysis was 27.55% (95% CI: [22.34; 32.77]) with 9422 positive cases from a total of 43,539 tested animals (Table 3, Supplementary Fig. S2), showing significant heterogeneity between studies (Q_71_ = 13206.68, *I*^*2*^ = 99.5%, *p* < 0.0001). This strong heterogeneity was additionally evidenced with a wide predictive interval [0.00-71.67%], demonstrating substantial differences between study variability in the seroprevalence estimates. Due to this high heterogeneity, a random effects model was used to calculate the pooled Q fever seroprevalence.

Subgroup analysis was performed to assess the contribution of species, country, detection method, sample size, and publication year for the seroprevalence of Q fever in animals (Table 3). According to species, camels were highly susceptible with a seroprevalence of 38.9% (95% CI: [24.60; 53.25]), followed by goats 38.0% (95% CI: [27.73; 48.23] and sheep 24.5% (95% CI: [16.48; 32.51]). Cattle were the least susceptible species with a seroprevalence of 14.6% (95% CI: [5.82; 23.37]). There was a statistically significant difference in Q fever seroprevalence between species (Q_3_ = 17.37, *p* = 0.0006) (Table 3, supplementary Fig. S3).

Regarding the country of studies, Q fever seroprevalence was higher in Sudan (38.9%, 95% CI: [0.00; 93.07]), followed by Ethiopia (31.5%, 95% CI: [22.77; 40.17]), Kenya (26.8%, 95% CI: [18.87; 34.74]), Tanzania (13.6%, 95% CI: [0.00; 31.60]), and lowest in Somalia (11.4%, 95% CI: [0.00; 38.79]) with a statistical significant variation between countries (Q_4_ = 12.35, *p* = 0.0149) (Table 3, Supplementary Fig. S4).

Q fever seroprevalence was assessed using different diagnostic methods resulting in higher seroprevalence when using ELISA (28.1%, 95% CI: [22.52; 33.72]) than IFA (22.4%, 95% CI: [5.01; 39.71]), with no statistically significant difference between detection methods (Q_1_= 0.57, *p* = 0.4498) (Table 3, Supplementary Fig. S5).

Among the study periods (categorized as 2012–2016, 2017–2021, and 2022–2025), seroprevalence of Q fever was higher in studies conducted between 2012 and 2016 (42.8%, 95% CI: [26.51; 59.04]) and lower between 2022 and 2025 (23.9%, 95% CI: [17.15; 30.54]), with no statistical significant difference (Q_2_ = 5.65, *p* = 0.0592) (Table 3, Supplementary Fig. S6).

Concerning sample size, we categorized the sample size into two groups (equal to or more than 384 and less than 384) and the result showed a higher seroprevalence of Q fever in studies with less than 384 samples (30.2%, 95% CI: [22.87; 37.52] (Table 3, Supplementary Fig. S7), without statistically significant difference (Q_1_= 1.72, *p* = 0.1895).

**Table 3:** The pooled seroprevalence of Q fever in animals

		No studies	No tested	No positive	Pooled estimate (95% CI)	Heterogeneity				Subgroup differences
						Q	*p*-value	I^2^ (%)	τ^2^	
Overall		72	43,539	9422	27.55 [22.34; 32.77]	13206.68	< 0.001	99.5	0.0483	
Species	Cattle	22	20,088	1868	14.6 [5.82; 23.37]	2049.29	< 0.001	99.0	0.0388	Q_3_ = 17.37; *p* = 0.0006
	Camel	12	2658	856	38.9 [24.60; 53.25]	574.97	< 0.001	98.1	0.0496	
	Sheep	18	8197	1896	24.5 [16.48; 32.51]	1136.76	< 0.001	98.5	0.0247	
	Goat	20	12,596	4802	38.0 [27.73; 48.23]	4788.93	< 0.001	99.6	0.0469	
Country	Ethiopia	25	9998	2600	31.5 [22.77; 40.17]	2452.20	< 0.001	99.0	0.0588	Q_4_ = 12.35; *p* = 0.0149
	Kenya	37	20,793	4894	26.8 [18.87; 34.74]	8778.98	< 0.001	99.6	0.0796	
	Sudan	3	780	231	38.9 [0.00; 93.07]	49.00	< 0.001	95.9	0.0478	
	Tanzania	4	11,596	1650	13.6 [0.00; 31.60]	966.28	< 0.001	99.7	0.0191	
	Somalia	3	372	47	11.4 [0.00; 38.79]	56.47	< 0.001	96.5	0.0524	
Detection method	ELISA	65	41,790	8865	28.1 [22.52; 33.72]	12174.28	< 0.001	99.5	0.0500	Q_1_ = 0.57; *p* = 0.4498
	IFA	7	1749	557	22.4 [5.01; 39.71]	888.24	< 0.001	99.3	0.0347	
Sample size	≥ 384	28	35,796	7261	23.6 [12.67; 26.74]	8500.39	< 0.001	99.7	0.0345	Q_1_ = 1.72; *p* = 0.1895
	< 384	44	7743	2161	30.2 [22.87; 37.52]	4585.23	< 0.001	99.1	0.0568	
Study period	2022–2025	38	35,048	7287	23.9 [17.15; 30.54]	9741.73	< 0.001	99.6	0.0406	Q_2_ = 5.65 *p* = 0.0592
	2017–2021	20	6110	1258	24.0 [16.17; 31.90]	1042.28	< 0.001	98.2	0.0268	
	2012–2016	14	2381	877	42.8 [26.51; 59.04]	1513.04	< 0.001	99.1	0.0781	

CI, confidence interval

### Pooled seroprevalence and subgroup meta-analysis of Q fever in humans

**Fig. 2:**
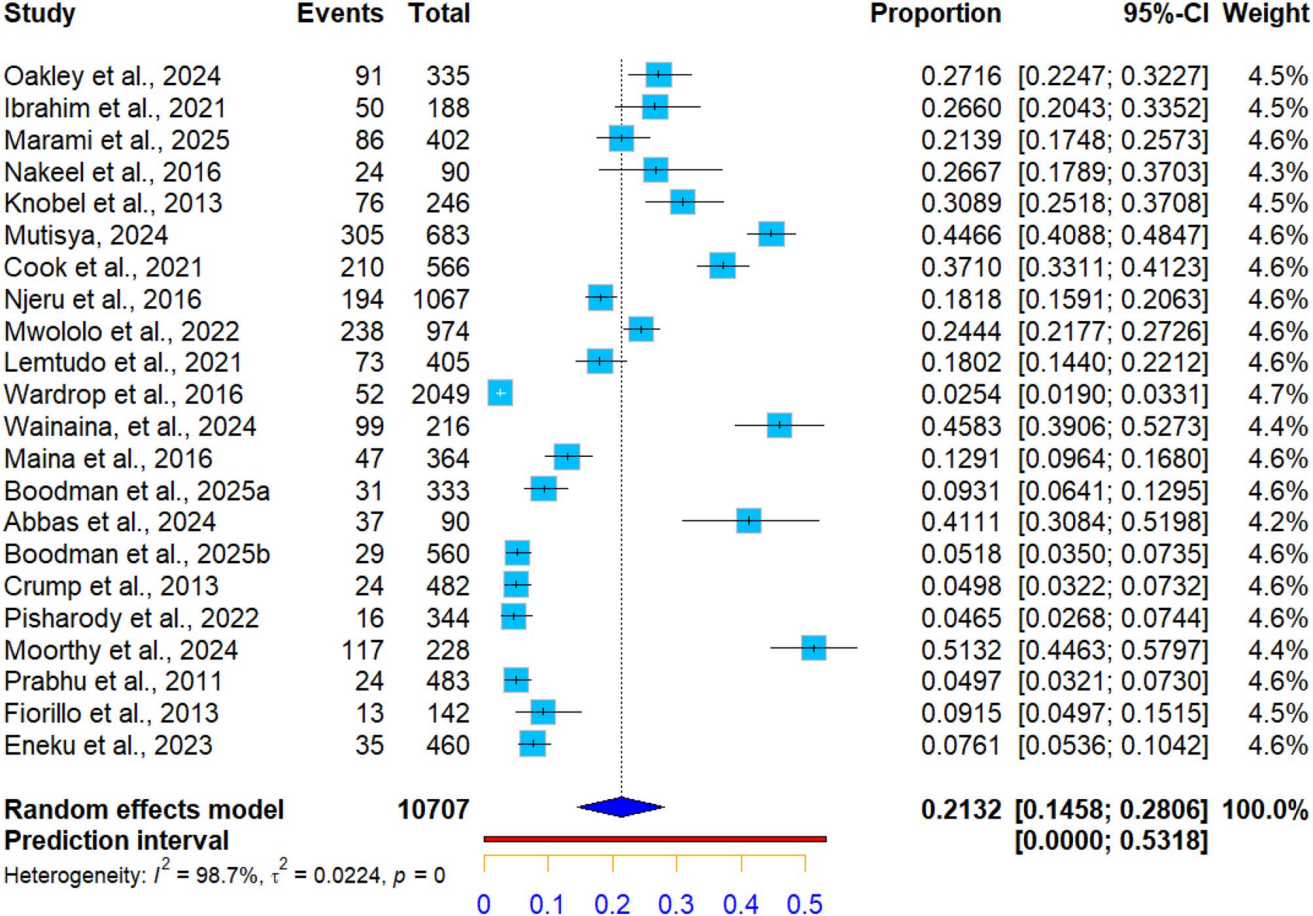
Forest plot showing pooled seroprevalence of Q fever in humans

The overall pooled Q fever seroprevalence in humans was 21.32% (95% CI: [14.58; 28.06]), with 1871 cases from 10,707 tested individuals. High level of heterogeneity was detected between studies (I^2^ = 98.7%, Q_21_ = 1664.85, *p* < 0.0001) (Table 4; Fig. 2).

The results of the subgroup analysis showed a significant variation in effect sizes in subgroups, including country and detection method. Among the countries, studies from Kenya (26.0%, 95% CI: [15.99; 35.95]) indicated the highest Q fever seroprevalence, while a study from Uganda (7.6%, 95% CI: [5.19; 10.03]) reported the lowest seroprevalence (Table 4, Supplementary Fig. S8). Subgroup differences between countries were statistically significant (Q_4_ = 64.70; *p* < 0.0001), with substantial heterogeneity ranging from 48.9% in Ethiopian studies to 99.3% in Kenyan studies, showing high study variability.

Concerning detection methods, pooled Q fever seroprevalence was similar for ELISA (21.4%, 95% CI: [13.64; 29.09]) and IFA (21.2%, 95% CI: [1.89; 40.58]) (Table 4, Supplementary Fig. S9). According to sample size category, there was no observed statistical significant difference in Q fever seroprevalence between studies with sample size less than 384 (25.7%, 95% CI: [15.08; 36.28]) and greater than or equal to 384 (17.1%, 95% CI: [7.69; 26.53]) (Table 4, Supplementary Fig. S10). Publication year was categorized into three groups, and higher Q fever seroprevalence was occurred in 2017–2021 studies (27.2%, 95% CI: [3.24; 51.23]) and 2022-2025studies (25.5%, 95% CI: [13.55; 37.34]) compared to pre-2017 studies (13.4%, 95% CI: [4.65; 22.08]) (Table 4, Supplementary Fig. S11). However, this difference was not statistically significant (Q_2_ = 3.47; *p* = 0.1760).

**Table 4:** The pooled seroprevalence of Q fever in humans

		No studies	No tested	No positive	Pooled estimate (95% CI)	Heterogeneity				Subgroup differences
						Q	*p*-value	I^2^ (%)	τ^2^	
Overall		22	10,707	1871	21.32 [14.58; 28.06]	1664.85	< 0.0001	98.7	0.0224	
Country	Ethiopia	3	925	227	24.7 [16.41; 32.98]	3.92	0.1411	48.9	0.0006	Q_4_ = 64.70; *p* < 0.0001
	Kenya	10	6660	1318	26.0 [15.99; 35.95]	1276.99	< 0.0001	99.3	0.0191	
	Sudan	3	983	97	18.0 [0.00; 66.32]	49.14	< 0.0001	95.9	0.0360	
	Tanzania	5	1679	194	14.9 [0.000; 40.00]	191.91	< 0.0001	97.9	0.0401	
	Uganda	1	460	35	7.6 [5.19; 10.03]	---	---	---	---	
Detection method	ELISA	16	8661	1511	21.4 [13.64; 29.09]	1351.71	< 0.0001	98.9	0.0203	Q_2_ = 0.0; *p* = 0.9874
	IFA	6	2046	360	21.2 [1.89; 40.58]	304.60	< 0.0001	98.4	0.0333	
Sample size	≥ 384	11	8131	1270	17.1 [7.69; 26.53]	1121.73	< 0.0001	99.1	0.0194	Q_1_ = 1.81; *p* = 0.1780
	< 384	11	2576	601	25.7 [15.08; 36.28]	404.75	< 0.0001	97.5	0.0241	
Publication year	2022–2025	11	4625	1084	25.5 [13.55; 37.34]	793.34	< 0.0001	98.7	0.0307	Q_2_ = 5.94; *p* = 0.0513
	2017–2021	3	1159	333	27.2 [3.24; 51.23]	46.85	< 0.0001	95.7	0.0089	
	2012–2016	8	4923	454	13.4 [4.65; 22.08]	295.73	< 0.0001	97.6	0.0101	

CI, confidence interval

### Publication bias and sensitivity analysis

**Fig. 3:**
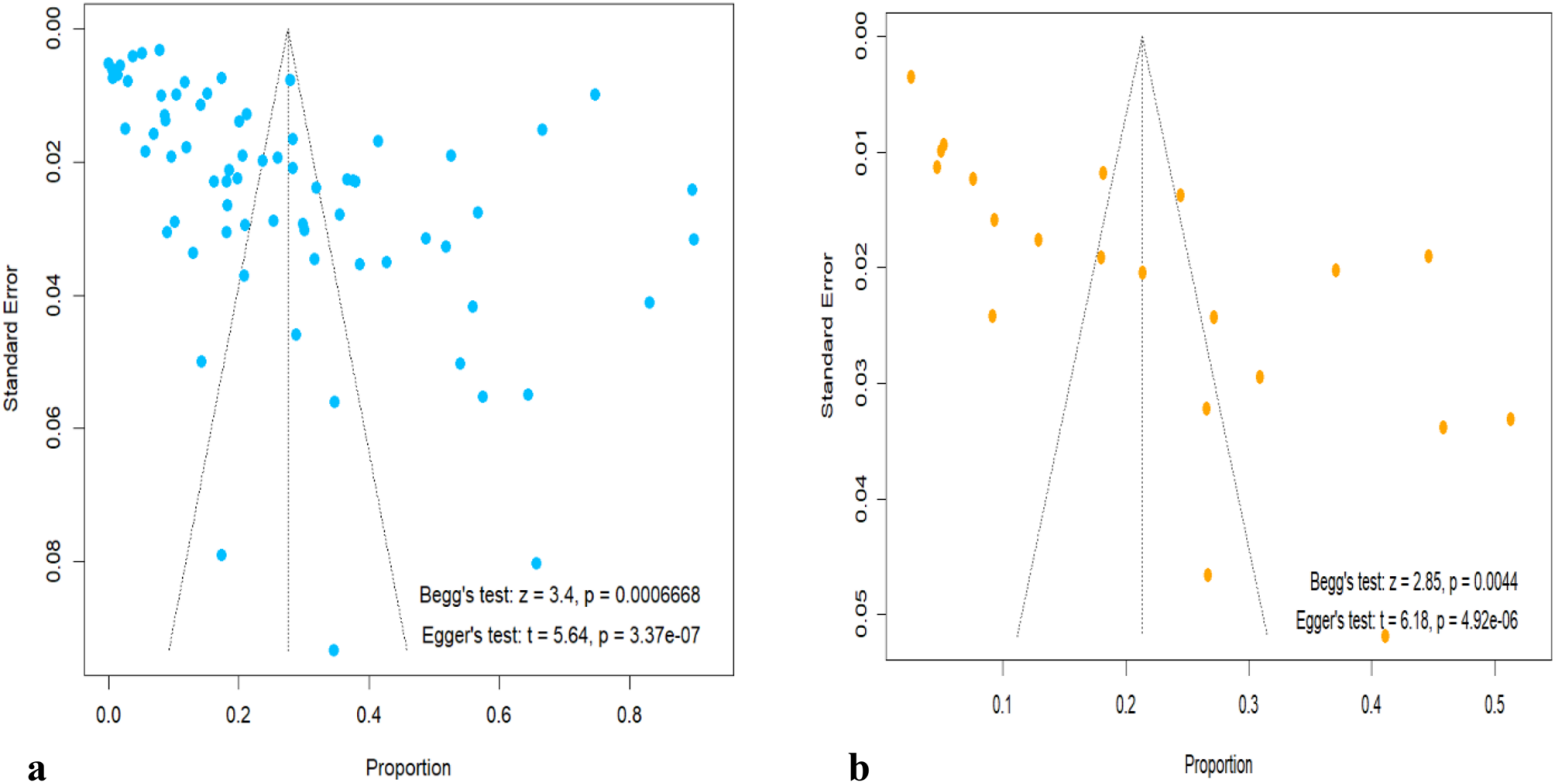
Funnel plot with pseudo 95% confidence interval to check publication bias of studies in animal (**a**) and humans (**b**)

Publication bias was assessed with funnel plots and using Begg’s and Egger’s tests (Fig. 3). The asymmetrical distributions observed in the funnel plot suggest that there was a possibility of publication bias in Q fever studies in animals. This was further supported by significant p-values of Begg’s (z = 3.4, *p* = 0.0007) and Egger’s (t = 5.64, *p* < 0.0001) tests, indicating statistically significant evidence of publication bias (Fig. 3a). According to the Duval and Tweedie’s trim-and-fill method, thirty-three studies were missed (Supplementary Fig. S12). After imputing the missed studies, the pooled seroprevalence was decreased from 27.55% (95% CI: [18.29; 27.95]) to 8.46% (95% CI: [1.67; 15.25]) with a prediction interval [0.00; 78.85], indicating the result was influenced by publication bias. The possibility of publication bias was also a concern in Q fever seroprevalence studies in humans with asymmetric distribution of the funnel plot and a significant Begg’s and Egger’s tests as shown in Fig. 3b. Ten studies were missing from Duval and Tweedie’s trim-and-fill method, and after imputing these missed studies (Supplementary Fig. S13), the pooled seroprevalence changed from 21.32% (95% CI: [14.58; 28.06]) to 6.98% (95% CI: [0.00; 15.90]) with a prediction interval [0.00; 59.99].

**Fig. 4:**
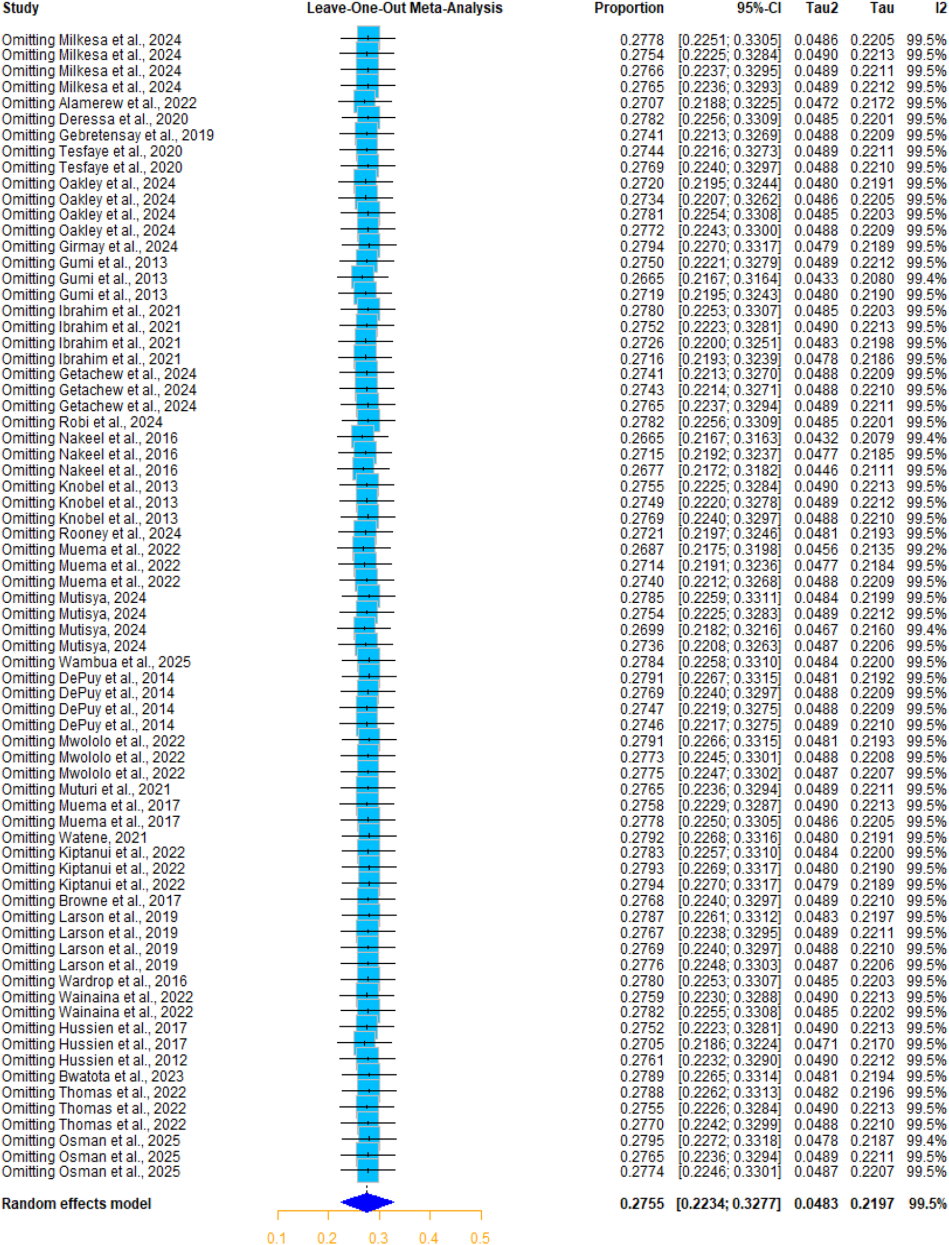
Leave-one-out meta-analysis of studies Q fever seroprevalence in animals

The sensitivity analysis using the Baujat diagnostic plot of animal studies indicated that studies by Thomas et al., 2022, Wambua et al., 2025, Bwatota et al., 2023, and Osman et al., 2025 contributed most to heterogeneity, and studies by Nakeel et al., 2016 in cattle and goats and Gumi et al., 2013 in camels influenced the pooled estimate and appeared to be potential outliers (Supplementary Fig. 14). However, the leave-one-out meta-analysis revealed no significant change in the pooled estimate, which ranged between 26.65% and 27.55%, indicating the robustness and reliability of the analysis (Fig. 4).

**Fig. 5:**
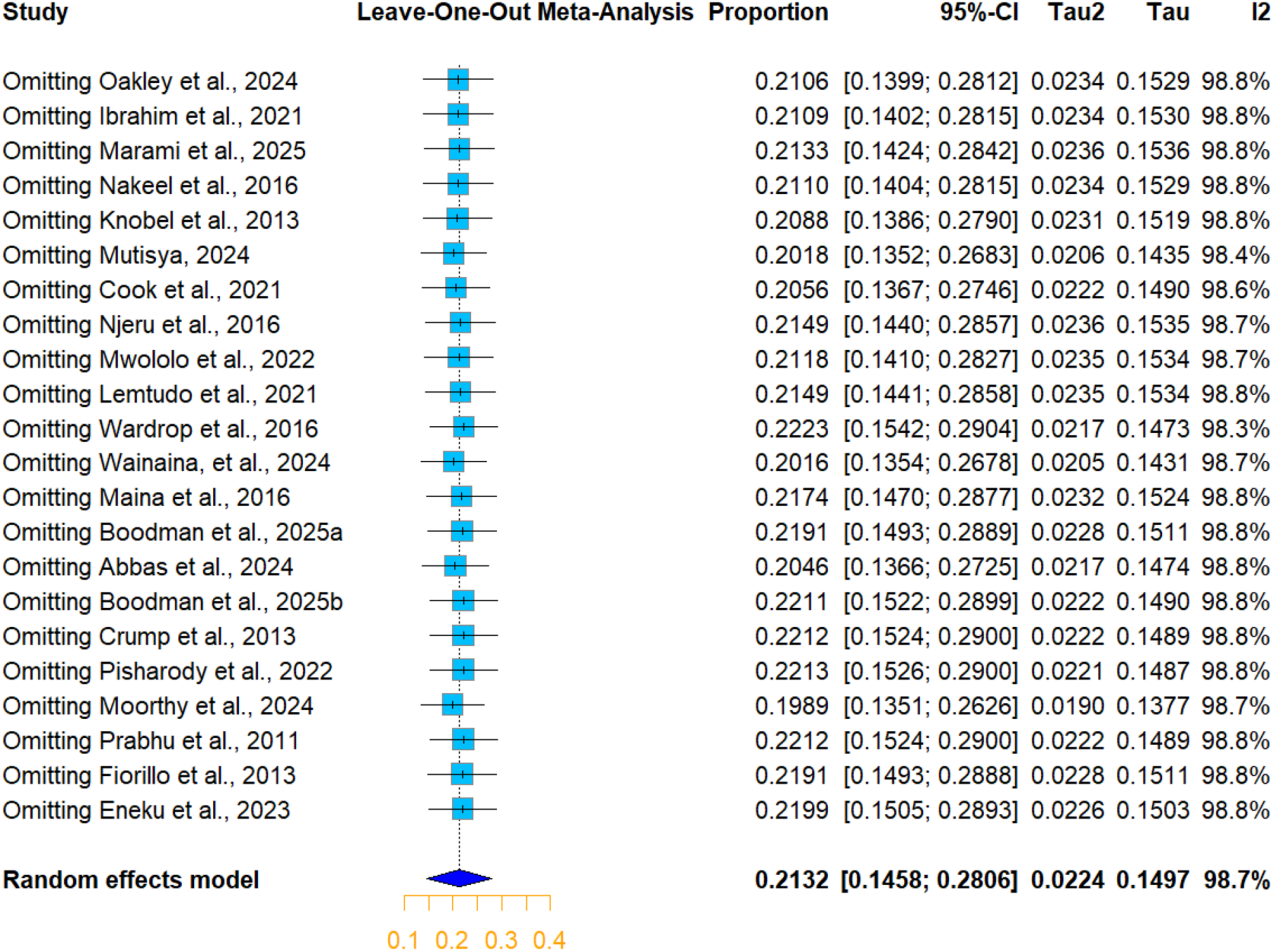
Leave-one-out meta-analysis of studies of Q fever seroprevalence in humans

In the human studies, although the Baujat plot (Supplementary Fig. S15) revealed that Moorthy et al., 2024 is the most influential study in the overall result, the sensitivity analysis by excluding one study at a time and recalculating the pooled seroprevalence showed no significant change, as demonstrated in the leave-one-out meta-analysis (Fig. 5). Furthermore, each study’s weight ranged between 4.2% and 4.7%, indicating the robustness and validity of the meta-analysis to the influence of individual study.

### Univariable and multivariable meta-regression analysis

In the univariable meta-regression analysis, species, country, and detection method were included as categorical predictors, while sample size and publication year were treated as continuous predictor variables.

The univariable meta-regression analysis showed a declining trend in Q fever seroprevalence in animals over time, with publication year accounting for 4.43% of the between-study variability. With regard to species, which explained 18.54% of the between-study variability, camel and goats had significantly higher Q fever seroprevalence compared to cattle, while there was no difference between sheep and cattle (Table 5). There was no observed difference in Q fever seroprevalence between countries or detection methods. In the multivariable meta-regression analysis, only species was the significant predictor variable. The final model explained 20.73% of the between-study variability (Table 5).

In human studies, univariable meta-regression showed Q fever seroprevalence increased with publication year, explaining 11.63% of the between-study variance. Country and detection method explained 0% of the between-study variance. Multivariable meta-regression analysis identified publication year, sample size, and country as significant predictors, with the overall model explaining 40.71% of the between-study variance (Table 6).

**Table 5:** Univariable and multivariable meta-regression analysis on Q fever prevalence in animals

Predictors	Categories	Univariable meta-regression				Multivariable meta-regression			
		R^2^ (%)	Estimate (95% CI)	SE	*p*	Estimate (95% CI)	SE	*p*	R^2^ (%)
Year of publication		4.43	-0.0137[-0.0270;-0.0004]	0.0067	0.0435	-0.0112 [-0.0239; 0.0015]	0.0063	0.0818	20.73
Sample size		1.31	0 [-0.0001; 0]	0.0	0.1621	0 [-0.0001; 0.000]	0.0	0.7470	
Species	Cattle	18.54	r			r			
	Camel		0.2431 [0.0993; 0.3869]	0.0721	0.0012	0.2310 [0.0851; 0.3768]	0.0730	0.0024	
	Goat		0.2336 [0.1101; 0.3570]	0.0619	0.0003	0.2241 [0.1014; 0.3468]	0.0614	0.0005	
	Sheep		0.0995 [-0.0274; 0.2264]	0.0636	0.1224	0.0959 [-0.0315; 0.2232]	0.0638	0.1375	
Country	Kenya	1.0	r						
	Ethiopia		0.0471 [-0.0669; 0.1612]	0.0571	0.4125				
	Somalia		-0.1510 [-0.4160; 0.1139]	0.1327	0.2592				
	Sudan		0.1217 [-0.1440; 0.3873]	0.1331	0.3639				
	Tanzania		-0.1320 [-0.3620; 0.0980]	0.1152	0.2560				
Detection method	ELISA	0	r						
	IFA		-0.0575 [-0.2342; 0.1192]	0.0886	0.5185				

R^2^, amount of heterogeneity accounted for, CI, confidence interval; r, reference category; SE, standard error

**Table 6:** Univariable and multivariable meta-regression analysis on Q fever seroprevalence in humans

Predictors	Categories	Univariable meta-regression				Multivariable meta-regression			
		R^2^ (%)	Estimate (95% CI)	SE	*p*	Estimate (95% CI)	SE	*p*	R^2^ (%)
Publication year		11.63	0.0128 [-0.0010; 0.0265]	0.0066	0.068	0.0186 [0.0036; 0.0337]	0.0071	0.019	40.71
Sample size		5.12	-0.0001 [-0.0003; 0.000]	0.0001	0.155	-0.0002 [-0.0003; 0.00]	0.0001	0.033	
Country	Kenya	0	r						
	Ethiopia		-0.0097 [-0.2269; 0.2076]	0.1029	0.926	-0.1491 [-0.3352; 0.0371]	0.0873	0.108	
	Sudan		-0.0822 [-0.3003; 0.1360]	0.1034	0.438	-0.2465 [-0.4403; -0.0528]	0.0909	0.016	
	Tanzania		-0.1125 [-0.2925; 0.0674]	0.0853	0.205	-0.1219 [-0.2743; 0.0304]	0.0715	0.109	
Detection method	ELISA	0	r						
	IFA		-0.0024 [-0.1578; 0.1529]	0.0745	0.975				

R^2^, amount of heterogeneity accounted for, CI, confidence interval; r, reference category, SE, standard error

### Trend of Q fever seroprevalence

**Fig. 6:**
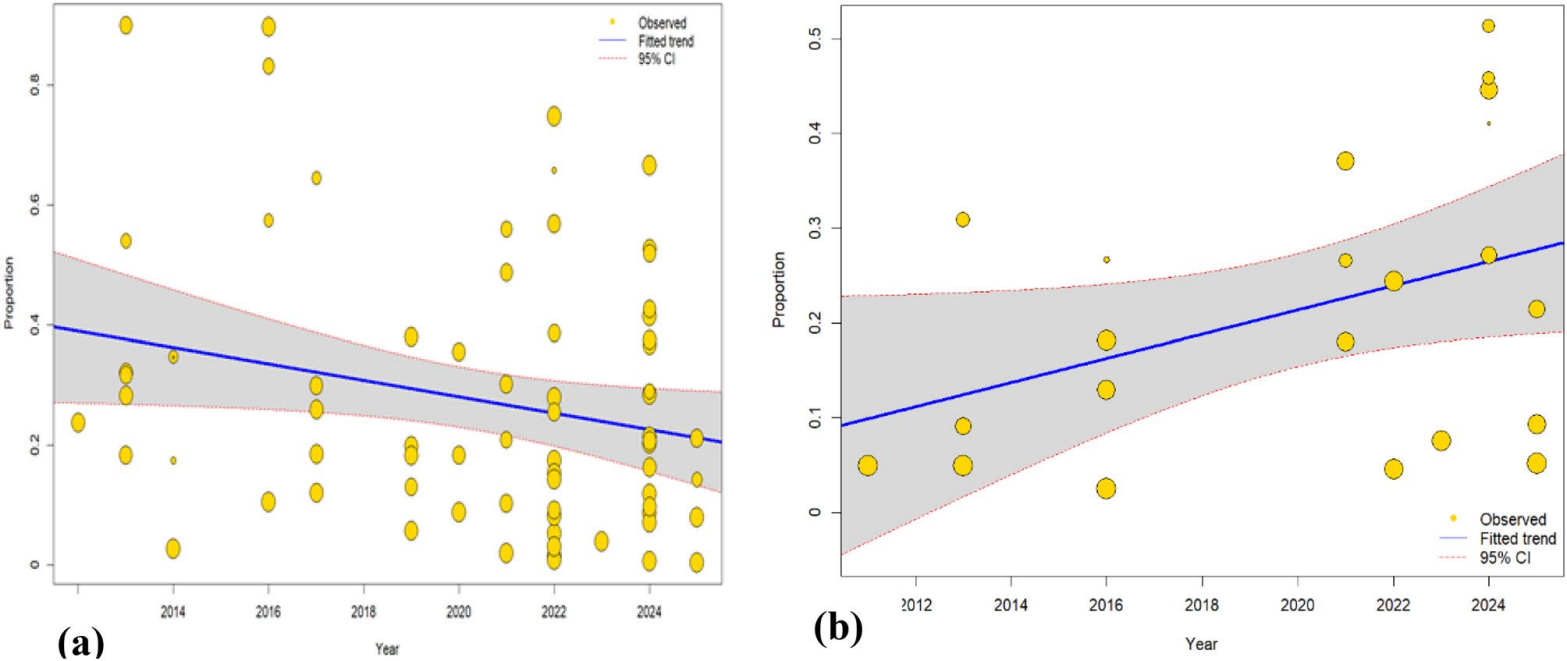
Yearly trend of Q fever seroprevalence in animals (**a**) in humans (**b**)

A random effects meta regression model was fitted to examine the presence of a temporal trend in effect sizes across studies. Q fever seroprevalence in animals showed a statistically significant temporal trend (Estimate = -0.0137, 95% CI: [-0.0267; -0.0006], *p* = 0.0398) decreasing over time (Fig. 6a). However, the estimated slope coefficient of the meta regression in humans was 0.0128 (SE = 0.0066), showing a positive but marginally non-significant increase (t = 1.9332, *p* = 0.0677) (Fig. 6b).

### Association between seroprevalence of Q fever and sex of the animals and humans

**Fig. 7:**
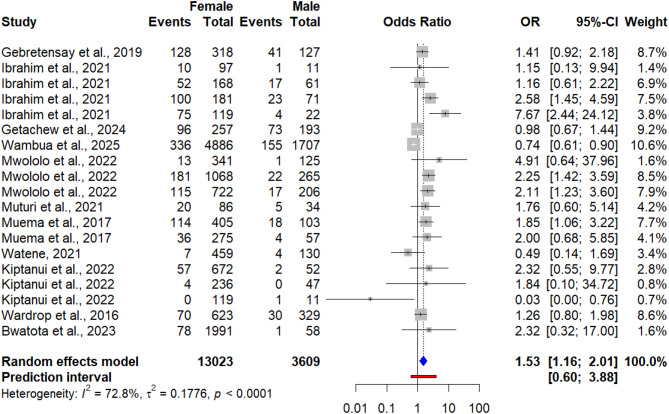
Forest plot of pooled odds ratio of sex and seroprevalence of Q fever in animals

**Fig. 8:**
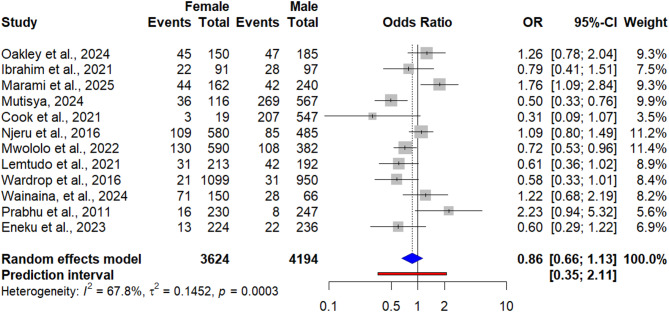
Forest plot of pooled odds ratio of gender and seroprevalence of Q fever in humans

Out of the total included studies, 22 reported outcomes with sex of the animal for each species. Therefore, the risk factor analysis regarding sex showed the odds of female animals contracting Q fever were about 1.52 times higher (OR = 1.52, 95% CI: 1.15; 2.01) than the odds in male animals (Fig. 7). However, in human studies, the result showed that Q fever seroprevalence was not significantly associated with gender (OR = 0.86, 95% CI: [0.66; 1.13]) (Fig. 8).

## Discussion

This study aimed to determine the overall seroprevalence of Q fever in animals and humans, assess the heterogeneity between studies, and check the trend of the pooled seroprevalence over time. In our systematic review and meta-analysis, 43,539 animals and 11,054 humans were tested, with 9422 positive animals and 1878 human positive cases in six East African countries (Ethiopia, Kenya, Sudan, Somalia, Tanzania, Uganda). The pooled estimate of Q fever was 27.55% and 21.32% in animals and humans, respectively. However, of the 13 East African countries included in the review, only six reported Q fever seroprevalence in humans and animals, indicating the insufficiency of data in the region.

The pooled seroprevalence of Q fever in animals in this study was comparable with the 22.6% report in the Eastern Mediterranean region [89], but it was higher compared to the 11.9% reported from South Asian countries [90]. In the current meta-analysis, Q fever seroprevalence was higher in camels and goats compared to sheep and cattle. The higher seroprevalence of Q fever in camels and goats might be attributable to the fact that camels and goats are predominantly reared in arid and semi-arid areas where environmental conditions may favor the survival and transmission of *C. burnetii*, with frequent exposure of the animals to sandstorms and dust. Additionally, camels’ broader-ranging behaviour may increase their exposure to tick-infested areas and contact with wildlife that may serve as reservoirs [91]. Goats are the most commonly marketed animals in the region, exposing them repeatedly to diseased animals at marketplaces and collection sites [92, 93]. Furthermore, goats are more prone to frequent kidding and have high placental shedding of *C. burnetii* during abortion events, which is a major source of environmental contamination and infection spread [94, 95].

The pooled seroprevalence of Q fever in camels was 38.9% with a report of individual studies ranging from 16.3% (Oakley et al., 2024) to 90.0% (Gumi et al., 2013) with both studies located in Ethiopia. The subgroup analysis of camel studies from three countries (Ethiopia, Kenya, and Sudan, conducted between 2013 and 2024) suggests they may be the main source of human Q fever infection. Comparable pooled seroprevalence of Q fever in camels was reported in Iran [96] and Eastern Mediterranean region [89]. In the current study, the seroprevalence of Q fever in goats was relatively higher with a pooled estimate of 38.0% covering five countries (Ethiopia, Kenya, Tanzania, Sudan, and Somalia). Higher (83.13%) [53] and lower (0.8%) [64] Q fever seroprevalence in goats was observed in Kenya, indicating high study variability even within countries. Similar pooled seroprevalence estimates were reported across other regions, including Oceania (29.54%), Africa (25.08%), Europe (25.23%), Asia (20.83%) [97], and Eastern Mediterranean region (28.15%) [89].

The pooled seroprevalence of Q fever in sheep was 24.5%, which ranged in individual studies from 1.4% [64] to 57.5% [53]. This result was in agreement with other finding from different regions such as 25.15% in Eastern Mediterranean region [89], 19.14% in Africa, 18.25% in Asia, 16.14% in Europe, and 15.04% in North America [97]. On the otherhand, the seroprevalence of Q fever in cattle was 14.6% ranging from 0.0% in Somalia [68] to 89.7% in Ethiopia [52]. Our finding was in agreement with previous reports of 20.16% in Eastern Mediterranean region [89] and 14.0% in Africa [22].

The subgroup analysis by country revealed that the seroprevalence of Q fever in animals was higher in Sudan (38.9%) and lower in Somalia (11.4%). However, the small number of studies in these countries, consisting of a sample size of 780 in Sudan and 372 in Somalia, indicates the need for further research to generate more robust evidence. Regarding the detection methods, the seroprevalence of Q fever in animals was higher in ELISA (28.1%) than in IFA (22.4%), although the difference was not statistically significant. The majority of the included studies used ELISA as a detection method, which is in line with other systematic review and meta-analysis reports [96–98]. Subgroup analysis by publication year showed decreasing Q fever seroprevalence in animals (42.8% before 2016, 24.0% in 2017–2021, and 23.9% after 2021). However, sample size varied greatly, with most of the studies (38 studies; 35,048 samples) being published after 2021.

The pooled seroprevalence of Q fever in humans in East Africa was 21.32%. Comparable findings were reported in different systematic review and meta-analysis studies from various regions, including the 26% in the Eastern Mediterranean region [89], 19.8% IgG phase I and 32.86% IgG phase II antibodies in Iran [96], 26% in abattoir and slaughterhouse workers globally [99], 16% in Africa [22]. However, the seroprevalence of Q fever in humans varies from region to region and between countries.

In the current meta-analysis, the highest pooled seroprevalence of Q fever in humans was recorded in Kenya (26.0%) ranging from 2.5% [67] to 45.8% [78] and Ethiopia (24.7%) ranging from 21.4% [74] to 27.2% [30]. In addition, seroprevalence of Q fever was reported in five studies in Tanzania with a seroprevalence ranging from 4.7% [84] to 51.3% [85] and three studies in Sudan ranging from 5.2% [80] to 41.1% [81]. A single study reported in Uganda with 7.6% Q fever seroprevalence [88], and it was excluded from the meta-regression analysis due to subgroups containing only one study cannot support variance estimation for effect size or moderator analysis [100]. In the multivariable meta-regression analysis, country was a significant predictor variable. The seroprevalence of Q fever in humans was higher in Kenya than Sudan, but it was similar with the seroprevalence in Ethiopia and Tanzania. The higher seroprevalence of Q fever in humans in Kenya, Ethiopia, and Tanzania might be due to, in addition to, the large pastoralist populations, rural communities, the practices of mixing herds and sharing watering resources, which increases transmission among animals and animals to humans, in these communities, risky behaviours are common including handling birth fluids and consumption of raw milk [30, 64]. The high seroprevalence of Q fever in humans in the meta-analysis might indicate the strong link of human and animals in East African countries.

According to detection methods, 16 studies used ELISA and six studies used IFA. Q fever seroprevalence was similar in those studies used ELISA (21.4%) and IFA (21.2%). The seroprevalence of Q fever in humans in this meta-analysis showed an increasing trend with publication year. The observed increase of Q fever in East Africa is primarily attributed to continuous high-risk exposures in pastoral communities, environmental persistence, absence of control programs, and intricate ecological and socio-economic factors that collectively perpetuate transmission cycles in the region. Pastoral communities are among the high risk groups that recorded high prevalence of Q fever. For example, Ibrahim et al. [101] and Oakley et al. [30] reported a seroprevalence of 27% in Somali and 25% in Afar pastoral regions of Ethiopia, respectively.

In the univariable analysis, Q fever seroprevalence showed a statistically decreasing trend over publication year in animals (*p* = 0.0307). However, multivariable adjustment for sample size and species revealed a non-significant trend, indicating persistently high Q fever seroprevalence in animals. Nowadays, Q fever had get more attention in research, but its impact continues in resource limited settings like East Africa. Unlike other developed countries, there are insufficient active Q fever control strategies and lack of a safe, effective and affordable Q fever vaccine in Africa [102, 103]. In addition, the continued high risk practices such as communal grazing and the agro-ecological conditions with periodic droughts and unbroken animal-tick transmission in the region keep animal infection rates relatively constant [22, 54].

In humans, the univariable meta-regression analysis showed that the seroprevalence of Q fever was increasing with publication year, but this was marginally non-significant. However, in the multivariable meta-regression analysis, after accounting for sample size and country, the trend was significantly increased over publication year (*p* = 0.019). Furthermore, there was no change on the statistical significance of the trend while the univariable and multivariable analysis was re-done by removing Moorthy et al. [85], which was indicated as a highly influential study based on the Baujat plot. This demonstrates model robustness and an increasing trend in human Q fever seroprevalence over time. This might indicate either the increasing of Q fever seroprevalence in humans in recent years or the number of seroprevalence surveys are increasing or enhanced diagnostic capabilities and getting research attention. However, routine clinical diagnosis remains severely limited and diagnostic challenges persist in the region. The predominant hot, arid, and semi-arid agro-ecological zones of East Africa coupled with the pastoral and agro-pastoral livestock system could create unique *C. burnetii* transmission dynamics. The pathogen’s spore-like form endures prolonged durations in arid, high-temperature conditions, while drought facilitates the aerial dissemination of contaminated dust [21, 104]. Among the included studies in our meta-analysis, the highest Q fever seroprevalence in humans was reported in pastoral communities and abattoir workers in Tanzania, Kenya, and Ethiopia [57, 75, 78, 85]. Although Q fever affects all social groups, studies confirm that high risk occupational groups including pastoralists, veterinarians, abattoir workers, herders, and farm workers are disproportionately affected [17, 105–107].

Out of the total included studies in the meta-analysis, only 11 studies assessed the association between animal sex and Q fever seroprevalence. Three studies (Mwololo et al., 2022; Muema et al., 2022; Larson et al., 2019) reported that female animals had higher odds of *C. burnetii* infection than male, while the remaining studies reported similar chance of *C. burnetii* infection. In our meta-analysis of all 11 included studies revealed that female animals had 1.53 times (OR = 1.53; 95% CI: [1.16; 2.01]) more likely to be positive to *C. burnetii* than males. This finding was in line with a recent meta-analysis on the global and regional seroprevalence of Q fever in small ruminants [97], where seroprevalence of Q fever was significantly higher in female animals than males. Similarly, several individual studies reported higher seroprevalence of Q fever in female animals [108–114]. The higher seroprevalence of Q fever in female animals could be due to *C. burnetii*’s strong tropism to reproductive tissues including uterus, mammary glands, and placenta [115, 116], from which it may be shed in subsequent parturitions and lactations. In addition, females are kept in production systems for longer periods for breeding and production resulting in prolonged exposure to contaminated environments, while males, on the other hand, are frequently sold at early age or raised separately, lowering their infection risk [92, 93]. However, other individual studies reported that female and male animals had similar odds of *C. burnetii* infection [117–119]. Unlike animals, our meta-analysis with 12 studies revealed men and women had equal chance of Q fever infection. This could be due to, in East African pastoral and rural communities, the overlap of occupational roles and routine domestic tasks which present exposure opportunities [67].

Despite indicating substantial Q fever burden across East Africa, these findings require cautious interpretation due to extreme heterogeneity (predominantly I^2^ > 98%), English-language restriction, geographic underrepresentation, and publication bias. The trim and fill analysis demonstrates profound publication bias, adjusting the seroprevalence downwards from 27.55% to 8.46% in animals and from 21.32% to 6.89% in humans. This > 3-fold bias adjustment underscores how selective reporting likely inflated seroprevalence estimates, highlighting the need for standardized surveillance and comprehensive study reporting across the region.

### Limitations

This systematic review and meta-analysis has several key limitations. Only English-language studies with accessible full-text were included, potentially excluding relevant seroprevalence data from non-English publications or subscription-restricted sources. Most publications originated from Kenya, Ethiopia, Tanzania, and Sudan, with limited representation from other East African countries, thereby restricting generalizability across the region. Additional limitations include extreme heterogeneity observed across subgroups due to diverse diagnostic methods and population characteristics undermine precise pooled seroprevalence estimates; strong evidence of publication bias (funnel plot asymmetry and significant statistical tests), likely inflating seroprevalence through underreporting of smaller/non-significant studies; over-reliance on serological methods (mostly ELISA) that detect past exposure rather than active *C. burnetii* infection; and the cross-sectional nature of most included studies, preventing robust assessment of temporal trends or intervention effects.

## Conclusion

The findings of this systematic review and meta-analysis indicate that Q fever is a significant zoonotic disease impacting both humans and animals in East Africa, with varying seroprevalence across different countries and host species. The disease is often under-recognized, primarily due to challenges in diagnosis and limited surveillance capacity. However, it poses substantial public health and economic risks, particularly for pastoralist communities that have close interactions with livestock. The identified data gaps in several countries underscore the pressing need for expanded and integrated One Health surveillance programs to better define the epidemiology and burden of Q fever in the region. Additionally, to reduce extreme heterogeneity and enable precise seroprevalence estimates, more research is required to be conducted in underrepresented East African countries using standardized diagnostic protocols. Such initiatives will be crucial for informing targeted control and prevention measures to alleviate the impact of this neglected disease on vulnerable populations and livestock production systems in East Africa.

## Supplementary Information

Below is the link to the electronic supplementary material.

Supplementary Material 1

Supplementary Material 2

## Data Availability

All the data used for analysis are available from the corresponding author upon reasonable request.

## Declarations

### Generative AI and AI-assisted technologies in the writing process

During the preparation of this work the authors used Grammarly and Perplexity in order to improve readability and language. After using this tool/service, the authors reviewed and edited the content as needed and take full responsibility for the content of the published article.

## Abbreviations

ELISA: Enzyme linked immunosorbent assay
JBI: Joanna Briggs Institute
IFA: Indirect immunofluorescence assay
PCR: Polymerase chain reaction
PRISMA: Preferred reporting items for systematic reviews and meta-analyses

## References

[1] Hechemy KE. History and Prospects of Coxiella burnetii Research. In: Toman R, Heinzen RA, Samuel JE, Mege J-L, editors. Coxiella Burn Recent Adv New Perspect Res Q Fever Bact [Internet]. Dordrecht: Springer Netherlands; 2012. pp. 1–11. 10.1007/978-94-007-4315-1_1. 10.1007/978-94-007-4315-1_1 22711624

[2] Cutler SJ, Bouzid M, Cutler RR. Q fever. J Infect. 2007;54:313–8. 10.1016/j.jinf.2006.10.048. 17147957 10.1016/j.jinf.2006.10.048

[3] WOAH. Q fever: chapter 3.1.18. Manual of diagnostic tests and vaccines for terrestrial animals. World Organization for Animal Health; 2019.

[4] Candela MG, Caballol A, Atance PM. Wide exposure to Coxiella burnetii in ruminant and feline species living in a natural environment: zoonoses in a human-livestock-wildlife interface. Epidemiol Infect. 2017;145:478–81. 10.1017/S0950268816002454. 27776577 10.1017/S0950268816002454 PMC9507642

[5] Devaux CA, Osman IO, Million M, Raoult D. Coxiella burnetii in Dromedary Camels (Camelus dromedarius): A Possible Threat for Humans and Livestock in North Africa and the Near and Middle East? Front Vet Sci. 2020;7:558481. 10.3389/fvets.2020.558481. 33251255 10.3389/fvets.2020.558481 PMC7674558

[6] Hussain S, Saqib M, El-Adawy H, Hussain MH, Jamil T, Sajid MS, et al. Seroprevalence and Molecular Evidence of Coxiella burnetii in Dromedary Camels of Pakistan. Front Vet Sci. 2022;9:908479. 10.3389/fvets.2022.908479. 35782546 10.3389/fvets.2022.908479 PMC9244431

[7] Pexara A, Solomakos N, Govaris A. Q fever and seroprevalence of Coxiella burnetii in domestic ruminants. Vet Ital. 2018;54:265–79. 10.12834/VetIt.1113.6046.3. 30681125 10.12834/VetIt.1113.6046.3

[8] Pouquet M, Bareille N, Guatteo R, Moret L, Beaudeau F. Coxiella burnetii infection in humans: to what extent do cattle in infected areas free from small ruminants play a role? Epidemiol Infect. 2020;148:e232. 10.1017/S0950268820001880. 32843112 10.1017/S0950268820001880 PMC7582459

[9] Agerholm JS. Coxiella burnetii associated reproductive disorders in domestic animals-a critical review. Acta Vet Scand. 2013;55:13. 10.1186/1751-0147-55-13. 23419216 10.1186/1751-0147-55-13 PMC3577508

[10] Burns RJL, Le KK, Siengsanun-Lamont J, Blacksell SD. A review of coxiellosis (Q fever) and brucellosis in goats and humans: Implications for disease control in smallholder farming systems in Southeast Asia. One Health. 2023;16:100568. 10.1016/j.onehlt.2023.100568. 37363211 10.1016/j.onehlt.2023.100568 PMC10288130

[11] Mutisya WM, Akoko JM, Mwatondo A, Muturi M, Nthiwa D, Abkallo HM, et al. Sero-epidemiology of Coxiella burnetii in livestock and humans in Isiolo county Kenya. PLoS Negl Trop Dis. 2025;19:e0013557. 10.1371/journal.pntd.0013557. 41105740 10.1371/journal.pntd.0013557 PMC12551958

[12] Ramo M, de los A, Benito AA, Quílez J, Monteagudo LV, Baselga C, Tejedor MT. Coxiella burnetii and Co-Infections with Other Major Pathogens Causing Abortion in Small Ruminant Flocks in the Iberian Peninsula. Anim Open Access J MDPI. 2022;12:3454. 10.3390/ani12243454. 10.3390/ani12243454 PMC9774532 36552374

[13] Robi DT, Demissie W, Temteme S. Coxiellosis in Livestock: Epidemiology, Public Health Significance, and Prevalence of Coxiella burnetii Infection in Ethiopia. Vet Med Auckl NZ. 2023;14:145–58. 10.2147/VMRR.S418346. 10.2147/VMRR.S418346 PMC10443632 37614223

[14] Eldin C, Mélenotte C, Mediannikov O, Ghigo E, Million M, Edouard S, et al. From Q Fever to Coxiella burnetii Infection: a Paradigm Change. Clin Microbiol Rev. 2017;30:115–90. 10.1128/CMR.00045-16. 27856520 10.1128/CMR.00045-16 PMC5217791

[15] Guatteo R, Seegers H, Taurel A-F, Joly A, Beaudeau F. Prevalence of Coxiella burnetii infection in domestic ruminants: a critical review. Vet Microbiol. 2011;149:1–16. 10.1016/j.vetmic.2010.10.007. 21115308 10.1016/j.vetmic.2010.10.007

[16] Anderson A, Boyer T, Garvey A, Marshall K, Menzies P, Murphy J, et al. Prevention and control of *Coxiella burnetii* infection among humans and animals: guidance for a coordinated public health and animal health response, 2013 [Internet]. 2013. https://www.nasphv.org/Documents/Q_Fever_2013.pdf.

[17] Groten T, Kuenzer K, Moog U, Hermann B, Maier K, Boden K. Who is at risk of occupational Q fever: new insights from a multi-profession cross-sectional study. BMJ Open. 2020;10:e030088. 10.1136/bmjopen-2019-030088. 32041851 10.1136/bmjopen-2019-030088 PMC7045227

[18] USAID. Improving livestock markets to generate economic growth and resilience in East Africa [Internet]. 2018 [cited 2025 Oct 24]. https://dai-global-developments.com/articles/improving-livestock-markets-to-generate-economic-growth-and-resilience-in-east-africa/. Accessed 24 Oct 2025.

[19] Georgiev M, Afonso A, Neubauer H, Needham H, Thiery R, Rodolakis A, et al. Q fever in humans and farm animals in four European countries, 1982 to 2010. Euro Surveill Bull Eur Sur Mal Transm Eur Commun Dis Bull. 2013;18:20407. 23449232

[20] Oketch DCO, Njoroge R, Ngage TO, Omar AA, Magarre A, Pasha R, et al. Cultural and behavioral drivers of zoonotic disease transmission and persistence among diverse pastoralist communities in East Africa. One Health Outlook. 2025;7:36. 10.1186/s42522-025-00153-9. 40660371 10.1186/s42522-025-00153-9 PMC12257696

[21] Kersh GJ, Fitzpatrick KA, Self JS, Priestley RA, Kelly AJ, Lash RR, et al. Presence and Persistence of Coxiella burnetii in the Environments of Goat Farms Associated with a Q Fever Outbreak. Appl Environ Microbiol. 2013;79:1697–703. 10.1128/AEM.03472-12. 23315737 10.1128/AEM.03472-12 PMC3591968

[22] Bwatota SF, Cook EAJ, Bronsvoort BM, de Wheelhouse C, Hernandez-Castro N, Shirima LE. GM. Epidemiology of Q-fever in domestic ruminants and humans in Africa: a systematic review. CAB International; 2022. [cited 2025 Oct 18]. 10.1079/cabionehealth.2022.0008.

[23] Christodoulou M, Malli F, Tsaras K, Billinis C, Papagiannis D. A Narrative Review of Q Fever in Europe. Cureus. 2023;15:e38031. 10.7759/cureus.38031. 37228530 10.7759/cureus.38031 PMC10207987

[24] Tan T, Heller J, Firestone S, Stevenson M, Wiethoelter A. A systematic review of global Q fever outbreaks. One Health. 2023;18:100667. 10.1016/j.onehlt.2023.100667. 39010957 10.1016/j.onehlt.2023.100667 PMC11247264

[25] Vanderburg S, Rubach MP, Halliday JEB, Cleaveland S, Reddy EA, Crump JA. Epidemiology of Coxiella burnetii Infection in Africa: A OneHealth Systematic Review. PLoS Negl Trop Dis Public Libr Sci. 2014;8:e2787. 10.1371/journal.pntd.0002787. 10.1371/journal.pntd.0002787 PMC3983093 24722554

[26] Bauer BU, Knittler MR, Herms TL, Frangoulidis D, Matthiesen S, Tappe D, et al. Multispecies Q fever outbreak in a mixed dairy goat and cattle farm based on a new bovine-associated genotype of *Coxiella burnetii*. Vet Sci [Internet]. Multidisciplinary Digital Publishing Institute; 2021. [cited 2026 Mar 14];8. 10.3390/vetsci8110252. 10.3390/vetsci8110252 PMC8626049 34822625

[27] Bond KA, Vincent G, Wilks CR, Franklin L, Sutton B, Stenos J, et al. One Health approach to controlling a Q fever outbreak on an Australian goat farm. Epidemiol Infect. 2016;144:1129–41. 10.1017/S0950268815002368. 26493615 10.1017/S0950268815002368 PMC4825098

[28] Meurer IR. The Importance of Medical Knowledge About Q Fever in the Context of Timely Diagnosis and Treatment and the Use of the One Health Approach in Combating This and Other Neglected Zoonotic Diseases [Letter]. Infect Drug Resist. 2025;18:5007–8. 10.2147/IDR.S567142. 41000263 10.2147/IDR.S567142 PMC12459374

[29] Munyua PM, Njenga MK, Osoro EM, Onyango CO, Bitek AO, Mwatondo A, et al. Successes and challenges of the One Health approach in Kenya over the last decade. BMC Public Health. 2019;19:465. 10.1186/s12889-019-6772-7. 32326940 10.1186/s12889-019-6772-7 PMC6696663

[30] Oakley RB, Gemechu G, Gebregiorgis A, Alemu A, Zinsstag J, Paris DH, et al. Seroprevalence and risk factors for Q fever and Rift Valley fever in pastoralists and their livestock in Afar, Ethiopia: A One Health approach. PLoS Negl Trop Dis. 2024;18:e0012392. 10.1371/journal.pntd.0012392. 39178328 10.1371/journal.pntd.0012392 PMC11376510

[31] Rahaman MR, Marshall H, Milazzo A, Crabb D, Bi P. Q fever prevention and vaccination: Australian livestock farmers’ knowledge and attitudes to inform a One Health approach. One Health. 2021;12:100232. 10.1016/j.onehlt.2021.100232. 33748388 10.1016/j.onehlt.2021.100232 PMC7960538

[32] Page MJ, McKenzie JE, Bossuyt PM, Boutron I, Hoffmann TC, Mulrow CD, et al. The PRISMA 2020 statement: An updated guideline for reporting systematic reviews. Int J Surg Lond Engl. 2021;88:105906. 10.1016/j.ijsu.2021.105906. 10.1016/j.ijsu.2021.105906 33789826

[33] Munn Z, Moola S, Lisy K, Riitano D, Tufanaru C. Methodological guidance for systematic reviews of observational epidemiological studies reporting prevalence and cumulative incidence data. JBI Evid Implement. 2015;13:147. 10.1097/XEB.0000000000000054. 10.1097/XEB.0000000000000054 26317388

[34] R Core Team. R: a language and environment for statistical computing. R Foundation for Statistical Computing, Vienna, Austria. [Internet]. 2025. https://www.R-project.org/.

[35] Higgins JPT, Thompson SG. Quantifying heterogeneity in a meta-analysis. Stat Med. 2002;21:1539–58. 10.1002/sim.1186. 12111919 10.1002/sim.1186

[36] IntHout J, Ioannidis JPA, Rovers MM, Goeman JJ. Plea for routinely presenting prediction intervals in meta-analysis. BMJ Open. 2016;6:e010247. 10.1136/bmjopen-2015-010247. 27406637 10.1136/bmjopen-2015-010247 PMC4947751

[37] Egger M, Davey Smith G, Schneider M, Minder C. Bias in meta-analysis detected by a simple, graphical test. BMJ. 1997;315:629–34. 10.1136/bmj.315.7109.629. 9310563 10.1136/bmj.315.7109.629 PMC2127453

[38] Begg CB, Mazumdar M. Operating characteristics of a rank correlation test for publication bias. Biometrics. 1994;50:1088–101. 7786990

[39] Baujat B, Mahé C, Pignon J-P, Hill C. A graphical method for exploring heterogeneity in meta-analyses: application to a meta-analysis of 65 trials. Stat Med. 2002;21:2641–52. 10.1002/sim.1221. 12228882 10.1002/sim.1221

[40] Viechtbauer W, Cheung MW-L. Outlier and influence diagnostics for meta-analysis. Res Synth Methods. 2010;1:112–25. 10.1002/jrsm.11. 26061377 10.1002/jrsm.11

[41] O’brien RM. A Caution Regarding Rules of Thumb for Variance Inflation Factors. Qual Quant. 2007;41:673–90. 10.1007/s11135-006-9018-6.

[42] Goodman LA, Kruskal WH. Measures of association for cross classifications. J Am Stat Assoc. [American Statistical Association, Taylor & Francis, Ltd.]; 1954;49:732–64. 10.2307/2281536.

[43] Milkesa A, Rufael T, Kinfe G, Belaineh R, Bulbula A, Cho D, et al. A sero-epidemiological analysis of Coxiella burnetii infection and its risk factors in livestock from Addis Ababa, Adama, and Modjo abattoirs and pastoral areas of Oromia, Ethiopia. PLoS Negl Trop Dis. 2024;18:e0012287. 10.1371/journal.pntd.0012287. 39012848 10.1371/journal.pntd.0012287 PMC11251603

[44] Alamerew EA, Yitagesu E, Areaya A, Aydefruhim D. Apparent prevalence of brucellosis, Q-fever and toxoplasmosis in aborted goat’s at North Shoa, Ethiopia. EUREKA Life Sci. 2022:28–37. 10.21303/2504-5695.2022.002611.

[45] Deressa FB, Kal DO, Gelalcha BD, Magalhães RJS. Seroprevalence of and risk factors for Q fever in dairy and slaughterhouse cattle of Jimma town, South Western Ethiopia. BMC Vet Res. 2020;16:385. 10.1186/s12917-020-02598-8. 33046069 10.1186/s12917-020-02598-8 PMC7552523

[46] Gebretensay A, Alemayehu G, Rekik M, Alemu B, Haile A, Rischkowsky B, et al. Risk factors for reproductive disorders and major infectious causes of abortion in sheep in the highlands of Ethiopia. Small Rumin Res. 2019;177:1–9. 10.1016/j.smallrumres.2019.05.019.

[47] Tesfaye A, Sahele M, Sori T, Guyassa C, Garoma A. Seroprevalence and associated risk factors for chlamydiosis, coxiellosis and brucellosis in sheep and goats in Borana pastoral area, southern Ethiopia. BMC Vet Res. 2020;16:145. 10.1186/s12917-020-02360-0. 32434500 10.1186/s12917-020-02360-0 PMC7238558

[48] Girmay G, Emeru BA, Tegegne DT, Bora SK, Gudeta WF, Dersso BS, et al. Seroprevalence of bovine Herpesvirus-1, bovine viral diarrhoea virus, Neospora caninum and Coxiella burnetii in dairy cows in Ethiopia. BMC Res Notes. 2024;17:394. 10.1186/s13104-024-07059-1. 39736785 10.1186/s13104-024-07059-1 PMC11684144

[49] Gumi B, Firdessa R, Yamuah L, Sori T, Tolosa T, Aseffa A, et al. Seroprevalence of brucellosis and Q-fever in southeast Ethiopian pastoral livestock. J Vet Sci Med Diagn. 2013;2. 10.4172/2325-9590.1000109. 10.4172/2325-9590.1000109 PMC3859921 24350302

[50] Ibrahim M, Schelling E, Zinsstag J, Hattendorf J, Andargie E, Tschopp R. Sero-prevalence of brucellosis, Q-fever and Rift Valley fever in humans and livestock in Somali Region, Ethiopia. PLoS Negl Trop Dis. 2021;15:e0008100. 10.1371/journal.pntd.0008100. 33493173 10.1371/journal.pntd.0008100 PMC7861547

[51] Getachew S, Kumsa B, Getachew Y, Kinfe G, Gumi B, Rufael T, et al. Seroprevalence of Coxiella burnetii and potential tick vectors infesting domestic ruminants and community perception of the disease in pastoral areas of south Omo zone, southern Ethiopia. Parasite Epidemiol Control. 2024;26:e00369. 10.1016/j.parepi.2024.e00369. 39131796 10.1016/j.parepi.2024.e00369 PMC11314887

[52] Robi DT, Bogale A, Urge B, Aleme M. Seroprevalence of Coxiella burnetii, Leptospira interrogans serovar hardjo, and Brucella species and associated reproductive disorders in cattle in southwest Ethiopia. Heliyon. 2024;10:e25558. 10.1016/j.heliyon.2024.e25558. 38327482 10.1016/j.heliyon.2024.e25558 PMC10848014

[53] Nakeel MJ, Arimi SM, Kitala PK, Nduhiu G, Njenga JM, Wabacha JK. A Sero-epidemiological Survey of Brucellosis, Q-Fever and Leptospirosis in Livestock and Humans and Associated Risk Factors in Kajiado County- Kenya. J Trop Dis [Internet]. 2016. 10.4172/2329-891X.1000215. [cited 2025 Sept 20];4.

[54] Knobel DL, Maina AN, Cutler SJ, Ogola E, Feikin DR, Junghae M, et al. Coxiella burnetii in humans, domestic ruminants, and ticks in rural western Kenya. Am J Trop Med Hyg. 2013;88:513–8. 10.4269/ajtmh.12-0169. 23382156 10.4269/ajtmh.12-0169 PMC3592534

[55] Rooney T, Fèvre EM, Villinger J, Brenn-White M, Cummings CO, Chai D, et al. Coxiella burnetii serostatus in dromedary camels (Camelus dromedarius) is associated with the presence of C. burnetii DNA in attached ticks in Laikipia County, Kenya. Zoonoses Public Health. 2024;71:503–14. 10.1111/zph.13127. 38627945 10.1111/zph.13127

[56] Muema J, Nyamai M, Wheelhouse N, Njuguna J, Jost C, Oyugi J, et al. Endemicity of Coxiella burnetii infection among people and their livestock in pastoral communities in northern Kenya. Heliyon. 2022;8:e11133. 10.1016/j.heliyon.2022.e11133. 36303929 10.1016/j.heliyon.2022.e11133 PMC9593183

[57] Mutisya WM. Estimation of seroprevalence and predictors for *Coxiella burnetii* infection in Garbatulla, Isiolo County, Kenya. [Internet] [Thesis]. University of Nairobi; 2024 [cited 2025 Sept 19]. http://erepository.uonbi.ac.ke/handle/11295/166897. Accessed 19 Sept 2025.

[58] Wambua L, Bett B, Abkallo HM, Muturi M, Nthiwa D, Nyamota R, et al. National serosurvey and risk mapping reveal widespread distribution of Coxiella burnetii in Kenya. Sci Rep. 2025;15:9706. 10.1038/s41598-025-94154-3. 40113846 10.1038/s41598-025-94154-3 PMC11926080

[59] DePuy W, Benka V, Massey A, Deem SL, Kinnaird M, O’Brien T, et al. Q Fever Risk Across a Dynamic, Heterogeneous Landscape in Laikipia County, Kenya. EcoHealth. 2014;11:429–33. 10.1007/s10393-014-0924-0. 24604546 10.1007/s10393-014-0924-0

[60] Mwololo D, Nthiwa D, Kitala P, Abuom T, Wainaina M, Kairu-Wanyoike S, et al. Sero-epidemiological survey of Coxiella burnetii in livestock and humans in Tana River and Garissa counties in Kenya. PLoS Negl Trop Dis. 2022;16:e0010214. 10.1371/journal.pntd.0010214. 35239658 10.1371/journal.pntd.0010214 PMC8923444

[61] Muturi M, Akoko J, Nthiwa D, Chege B, Nyamota R, Mutiiria M, et al. Serological evidence of single and mixed infections of Rift Valley fever virus, Brucella spp. and Coxiella burnetii in dromedary camels in Kenya. PLoS Negl Trop Dis. 2021;15:e0009275. 10.1371/journal.pntd.0009275. 33770095 10.1371/journal.pntd.0009275 PMC7997034

[62] Muema J, Thumbi SM, Obonyo M, Wanyoike S, Nanyingi M, Osoro E, et al. Seroprevalence and Factors Associated with Coxiella burnetii Infection in Small Ruminants in Baringo County, Kenya. Zoonoses Public Health. 2017;64:e31–43. 10.1111/zph.12342. 28117947 10.1111/zph.12342

[63] Watene GAW. Sero-prevalence and risk factors associated with *Coxiella burnetii* from cattle in the Masaai Mara ecosystem in Narok, Kenya [Internet] [Thesis]. UON; 2021 [cited 2025 Sept 19]. http://erepository.uonbi.ac.ke/handle/11295/160672. Accessed 19 Sept 2025.

[64] Kiptanui J, Gathura PB, Kitala PM, Bett B. Seroprevalence Estimates of Q Fever and the Predictors for the Infection in Cattle, Sheep, and Goats in Nandi County, Kenya. Vet Med Int. 2022;2022:3741285. 10.1155/2022/3741285. 36437838 10.1155/2022/3741285 PMC9683944

[65] Browne AS, Fèvre EM, Kinnaird M, Muloi DM, Wang CA, Larsen PS, et al. Serosurvey of Coxiella burnetii (Q fever) in Dromedary Camels (Camelus dromedarius) in Laikipia County, Kenya. Zoonoses Public Health. 2017;64:543–9. 10.1111/zph.12337. 28176495 10.1111/zph.12337 PMC5655913

[66] Larson PS, Espira L, Grabow C, Wang CA, Muloi D, Browne AS, et al. The sero-epidemiology of Coxiella burnetii (Q fever) across livestock species and herding contexts in Laikipia County, Kenya. Zoonoses Public Health. 2019;66:316–24. 10.1111/zph.12567. 30788910 10.1111/zph.12567 PMC6563451

[67] Wardrop NA, Thomas LF, Cook EAJ, de Glanville WA, Atkinson PM, Wamae CN, et al. The Sero-epidemiology of Coxiella burnetii in Humans and Cattle, Western Kenya: Evidence from a Cross-Sectional Study. PLoS Negl Trop Dis. 2016;10:e0005032. 10.1371/journal.pntd.0005032. 27716804 10.1371/journal.pntd.0005032 PMC5055308

[68] Osman AM, Hassan-Kadle AA, Silito IS, Secato CT, Ibrahim AM, Serpa MCA, et al. Coxiella burnetii in ruminants from Somalia. Trop Anim Health Prod. 2025;57:381. 10.1007/s11250-025-04649-4. 40982092 10.1007/s11250-025-04649-4 PMC12454529

[69] Wainaina M, Lindahl JF, Dohoo I, Mayer-Scholl A, Roesel K, Mbotha D, et al. Longitudinal Study of Selected Bacterial Zoonoses in Small Ruminants in Tana River County, Kenya. Microorganisms Multidisciplinary Digit Publishing Inst. 2022;10:1546. 10.3390/microorganisms10081546. 10.3390/microorganisms10081546 PMC9414833 36013964

[70] Hussien MO, Enan KA, Hassan Alfaki S, Alhibir Gafar R, Mohamed Taha K. Rahim Mohamed El Hussein A. Seroprevalence of Coxiella burnetii in Dairy Cattle and Camel in Sudan. Int J Infect [Internet]. 2016. 10.5812/iji.42945. [cited 2025 Sept 19];4.

[71] Hussien M, ElFahal A, Enan K, Taha K, Mohammed M, Salih D, et al. Seroprevalence of Q fever in Goats in the Sudan. Vet World. 2012;5:394. 10.5455/vetworld.2012.394-397.

[72] Bwatota SF, Shirima GM, Hernandez-Castro LE, Bronsvoort BM, de Wheelhouse C, Mengele N. Seroprevalence and Risk Factors for Q fever (Coxiella burnetii) Exposure in Smallholder Dairy Cattle in Tanzania. Vet Sci. 2022;9:662. 10.3390/vetsci9120662. 36548823 10.3390/vetsci9120662 PMC9784148

[73] Thomas KM, Kibona T, Claxton JR, de Glanville WA, Lankester F, Amani N, et al. Prospective cohort study reveals unexpected aetiologies of livestock abortion in northern Tanzania. Sci Rep Nat Publishing Group. 2022;12:11669. 10.1038/s41598-022-15517-8. 10.1038/s41598-022-15517-8 PMC9270399 35803982

[74] Marami D, Mihret A, Assefa N, Abdissa A, Osman M, Gemechu G, et al. Rift Valley fever virus and Coxiella burnetii infections among febrile patients, Eastern Ethiopia. PLoS Negl Trop Dis. 2025;19:e0013375. 10.1371/journal.pntd.0013375. 40802844 10.1371/journal.pntd.0013375 PMC12364338

[75] Cook EAJ, de Glanville WA, Thomas LF, Kiyong’a A, Kivali V, Kariuki S, et al. Evidence of exposure to *C. burnetii* among slaughterhouse workers in western Kenya. One Health. 2021;13:100305. 10.1016/j.onehlt.2021.100305. 34430697 10.1016/j.onehlt.2021.100305 PMC8367830

[76] Njeru J, Henning K, Pletz MW, Heller R, Forstner C, Kariuki S, et al. Febrile patients admitted to remote hospitals in Northeastern Kenya: seroprevalence, risk factors and a clinical prediction tool for Q-Fever. BMC Infect Dis. 2016;16:244. 10.1186/s12879-016-1569-0. 27260261 10.1186/s12879-016-1569-0 PMC4891891

[77] Lemtudo AP, Mutai BK, Mwamburi L, Waitumbi JN. Seroprevalence of Coxiella burnetii in patients presenting with acute febrile illness at Marigat District Hospital, Baringo County, Kenya. Vet Med Sci. 2021;7:2093–9. 10.1002/vms3.493. 33955713 10.1002/vms3.493 PMC8464244

[78] Wainaina M, Lindahl JF, Mayer-Scholl A, Ufermann C-M, Domelevo Entfellner J-B, Roesler U, et al. Molecular and serological diagnosis of multiple bacterial zoonoses in febrile outpatients in Garissa County, north-eastern Kenya. Sci Rep Nat Publishing Group. 2024;14:12263. 10.1038/s41598-024-62714-8. 10.1038/s41598-024-62714-8 PMC11133362 38806576

[79] Maina AN, Farris CM, Odhiambo A, Jiang J, Laktabai J, Armstrong J, et al. Q Fever, Scrub Typhus, and Rickettsial Diseases in Children, Kenya, 2011–2012. Emerg Infect Dis. 2016;22:883–6. 10.3201/eid2205.150953. 27088502 10.3201/eid2205.150953 PMC4861507

[80] Boodman C, Edouard S, van Griensven J, Koirala KD, Khanal B, Rijal S, et al. *Coxiella burnetii* and *Bartonella* species serology of febrile patients with an established infectious or inflammatory diagnosis in Sudan, Nepal, and Cambodia. Microbiol Spectr [Internet]. American Society for Microbiology1752 N St., N.W., Washington DC et al. 2025 [cited 2025 Sept 24]; 10.1128/spectrum.01675-25. Accessed 24 Sept 2025. 10.1128/spectrum.01675-25 PMC12584713 40970723

[81] Abbas SSEM, Ahmed MYA, Abdamalik AHE, Suliman AJE, Yousef AAA, Abdudafea AOM, et al. Sero-Detection of Coxiella Burnett (Q fever) Infection Anti-Antibodies Igg and Igm among Spontaneous, Recurrent Miscarriage Women in Gezira State, Sudan -Case-Control Studies 2018. Middle East Res J Med Sci. 2024;4:70–5. 10.36348/merjms.2024.v04i04.001.

[82] Boodman C, Edouard S, van Griensven J, Koirala KD, Khanal B, Rijal S, et al. Evidence of Coxiella burnetii and Bartonella species infections among patients with persistent febrile illness in four low- and middle-income countries. Clin Microbiol Infect Off Publ Eur Soc Clin Microbiol Infect Dis. 2025;31:1389–93. 10.1016/j.cmi.2025.04.038. 10.1016/j.cmi.2025.04.038 40339791

[83] Crump JA, Morrissey AB, Nicholson WL, Massung RF, Stoddard RA, Galloway RL, et al. Etiology of Severe Non-malaria Febrile Illness in Northern Tanzania: A Prospective Cohort Study. PLoS Negl Trop Dis Public Libr Sci. 2013;7:e2324. 10.1371/journal.pntd.0002324. 10.1371/journal.pntd.0002324 PMC3715424 23875053

[84] Pisharody S, Rubach MP, Carugati M, Nicholson WL, Perniciaro JL, Biggs HM, et al. Incidence Estimates of Acute Q Fever and Spotted Fever Group Rickettsioses, Kilimanjaro, Tanzania, from 2007 to 2008 and from 2012 to 2014. Am J Trop Med Hyg. 2022;106:494–503. 10.4269/ajtmh.20-1036. 10.4269/ajtmh.20-1036 PMC8832940 34929672

[85] Moorthy GS, Rubach MP, Maze MJ, Refuerzo RP, Shirima GM, Lukambagire AS, et al. Prevalence and risk factors for Q fever, spotted fever group rickettsioses, and typhus group rickettsioses in a pastoralist community of northern Tanzania, 2016–2017. Trop Med Int Health TM IH. 2024;29:365–76. 10.1111/tmi.13980. 38480005 10.1111/tmi.13980 PMC11073910

[86] Prabhu M, Nicholson WL, Roche AJ, Kersh GJ, Fitzpatrick KA, Oliver LD, et al. Q fever, spotted fever group, and typhus group rickettsioses among hospitalized febrile patients in northern Tanzania. Clin Infect Dis Off Publ Infect Dis Soc Am. 2011;53:e8–15. 10.1093/cid/cir411. 10.1093/cid/cir411 PMC3148261 21810740

[87] Fiorillo SP, Diefenthal HC, Goodman PC, Ramadhani HO, Njau BN, Morrissey AB, et al. Chest radiography for predicting etiology of febrile illness among inpatients in Moshi, Tanzania. Clin Radiol. 2013;68:1039–46. 10.1016/j.crad.2013.05.002. 23809268 10.1016/j.crad.2013.05.002 PMC3759645

[88] Eneku W, Erima B, Byaruhanga AM, Cleary NG, Atim G, Tugume T, et al. Seroprevalence of Q-fever, spotted fever, typhus group *Rickettsia* and *Orientia* among febrile patients visiting hospital-based sentinel sites in Uganda: a cross-sectional study. PAMJ-One Health [Internet]. 2023 [cited 2025 Sept 19];12. 10.11604/pamj-oh.2023.12.3.41475.

[89] Ahmadinezhad M, Mounesan L, Doosti-Irani A, Behzadi MY. The prevalence of Q fever in the Eastern Mediterranean region: a systematic review and meta-analysis. Epidemiol Health. 2022;44:e2022097. 10.4178/epih.e2022097. 36317399 10.4178/epih.e2022097 PMC10396516

[90] Islam MM, Dutta P, Bansal D, Gongal G, Farag E, Magalhaes RJS, et al. Prevalence and Risk Factors of Coxiellosis at the Human-Animal-Environment Interface in the South Asian Countries: A Systematic Review and Meta-Analysis. Transbound Emerg Dis. 2025;2025:2890693. 10.1155/tbed/2890693. 40302760 10.1155/tbed/2890693 PMC12016896

[91] Celina SS, Cerný J. Coxiella burnetii in ticks, livestock, pets and wildlife: A mini-review. Front Vet Sci [Internet] Front. 2022. 10.3389/fvets.2022.1068129. [cited 2025 Nov 10];9. 10.3389/fvets.2022.1068129 PMC9691889 36439350

[92] Kichamu N, Astuti PK, Wanjala G, Strausz P, Bagi Z, Kusza S. A Review on Indigenous Goats of East Africa: A Case for Conservation and Management. Biology. 2024;13:419. 10.3390/biology13060419. 38927299 10.3390/biology13060419 PMC11200369

[93] Tschopp R, GebreGiorgis A, Abdulkadir O, Molla W, Hamid M, Tassachew Y, et al. Risk factors for Brucellosis and knowledge-attitude practice among pastoralists in Afar and Somali regions of Ethiopia. Prev Vet Med. 2022;199:105557. 10.1016/j.prevetmed.2021.105557. 34902652 10.1016/j.prevetmed.2021.105557

[94] de Cremoux R, Rousset E, Touratier A, Audusseau G, Nicollet P, Ribaud D, et al. Coxiella burnetii vaginal shedding and antibody responses in dairy goat herds in a context of clinical Q fever outbreaks. FEMS Immunol Med Microbiol. 2012;64:120–2. 10.1111/j.1574-695X.2011.00893.x. 22066517 10.1111/j.1574-695X.2011.00893.x

[95] Muleme M, Campbell A, Stenos J, Devlin JM, Vincent G, Cameron A, et al. A longitudinal study of serological responses to Coxiella burnetii and shedding at kidding among intensively-managed goats supports early use of vaccines. Vet Res. 2017;48:50. 10.1186/s13567-017-0452-3. 28915918 10.1186/s13567-017-0452-3 PMC5603018

[96] Mobarez AM, Amiri FB, Esmaeili S. Seroprevalence of Q fever among human and animal in Iran; A systematic review and meta-analysis. PLoS Negl Trop Dis. 2017;11:e0005521. 10.1371/journal.pntd.0005521. 28394889 10.1371/journal.pntd.0005521 PMC5398711

[97] Ahaduzzaman M, Reza MMB. Global and regional seroprevalence of coxiellosis in small ruminants: A systematic review and meta-analysis. Vet Med Sci. 2024;10:e1441. 10.1002/vms3.1441. 38613179 10.1002/vms3.1441 PMC11015088

[98] Konputtar A, Nam NH, Rerkyusuke S, Thamrongyoswittayakul C, Seesupa S, Yossapol M, et al. Herd-level seroprevalence, molecular prevalence, and trends of Coxiella burnetii (Q fever) in cattle worldwide: A systematic review and meta-analysis. Vet World. 2024;17:2811–28. 10.14202/vetworld.2024.2811-2828. 39897364 10.14202/vetworld.2024.2811-2828 PMC11784057

[99] Woldeyohannes SM, Gilks CF, Baker P, Perkins NR, Reid SA. Seroprevlance of Coxiella burnetii among abattoir and slaughterhouse workers: A meta-analysis. One Health Amst Neth. 2018;6:23–8. 10.1016/j.onehlt.2018.09.002. 10.1016/j.onehlt.2018.09.002 PMC6175780 30302365

[100] Cuijpers P, Griffin JW, Furukawa TA. The lack of statistical power of subgroup analyses in meta-analyses: a cautionary note. Epidemiol Psychiatr Sci. 2021;30:e78. 10.1017/S2045796021000664. 34852862 10.1017/S2045796021000664 PMC8679832

[101] Ibrahim M, Schelling E, Zinsstag J, Hattendorf J, Andargie E, Tschopp R. Sero-prevalence of brucellosis, Q-fever and Rift Valley fever in humans and livestock in Somali Region, Ethiopia. PLoS Negl Trop Dis. 2021;15:e0008100. 10.1371/journal.pntd.0008100. 33493173 10.1371/journal.pntd.0008100 PMC7861547

[102] McNeilly TN. Q fever: an emerging problem in LMIC and the need for improved vaccines [Internet]. Open Access Gov. 2019 [cited 2025 Oct 29]. https://www.openaccessgovernment.org/improved-vaccines/62803/. Accessed 29 Oct 2025.

[103] Njeru J, Henning K, Pletz MW, Heller R, Neubauer H. Q fever is an old and neglected zoonotic disease in Kenya: a systematic review. BMC Public Health. 2016;16:297. 10.1186/s12889-016-2929-9. 27048480 10.1186/s12889-016-2929-9 PMC4822290

[104] Van Leuken JPG, Swart AN, Brandsma J, Terink W, Van de Kassteele J, Droogers P, et al. Human Q fever incidence is associated to spatiotemporal environmental conditions. One Health. 2016;2:77–87. 10.1016/j.onehlt.2016.03.004. 28616479 10.1016/j.onehlt.2016.03.004 PMC5441340

[105] Fincato A, Lucchese L, Bellinati L, Mazzotta E, Ragolia S, Asa’Ad S, et al. Q Fever: Who Is at Risk? A Serological Survey in the General Population and Occupationally Exposed Individuals in Northern Italy. Pathogens. 2025;14:869. 10.3390/pathogens14090869. 41011769 10.3390/pathogens14090869 PMC12472743

[106] Özcelik R, Abakar MF, Counotte MJ, Abdelrazak Zakaria F, Kimala P, Issa R, et al. Seroprevalence and associated risk factors of brucellosis, Rift Valley fever and Q fever among settled and mobile agro-pastoralist communities and their livestock in Chad. PLoS Negl Trop Dis. 2023;17:e0011395. 10.1371/journal.pntd.0011395. 37352362 10.1371/journal.pntd.0011395 PMC10351688

[107] Simpson GJG, Quan V, Frean J, Knobel DL, Rossouw J, Weyer J, et al. Prevalence of selected zoonotic diseases and risk factors at a human-wildlife-livestock interface in Mpumalanga Province, South Africa. Vector-Borne Zoonotic Dis. Mary Ann Liebert, Inc., publishers; 2018;18:303–10. 10.1089/vbz.2017.2158. 10.1089/vbz.2017.2158 29664701

[108] Abakar MF, Naré NB, Schelling E, Hattendorf J, Alfaroukh IO, Zinsstag J. Seroprevalence of Rift Valley fever, Q fever, and brucellosis in ruminants on the southeastern shore of Lake Chad. Vector Borne Zoonotic Dis Larchmt N. 2014;14:757–62. 10.1089/vbz.2014.1585. 10.1089/vbz.2014.1585 25325320

[109] Esmaeili S, Mostafavi E, Shahdordizadeh M, Mahmoudi H. A seroepidemiological survey of Q fever among sheep in Mazandaran province, northern Iran. Ann Agric Environ Med AAEM. 2013;20:708–10. 24364439

[110] Mangena M, Gcebe N, Pierneef R, Thompson PN, Adesiyun AA. Q Fever: Seroprevalence, Risk Factors in Slaughter Livestock and Genotypes of Coxiella burnetii in South Africa. Pathog Basel Switz. 2021;10:258. 10.3390/pathogens10030258. 10.3390/pathogens10030258 PMC7996333 33668366

[111] Sakhaee E, Khalili M. The first serologic study of Q fever in sheep in Iran. Trop Anim Health Prod. 2010;42:1561–4. 10.1007/s11250-010-9606-2. 20521106 10.1007/s11250-010-9606-2

[112] Siengsanan-Lamont J, Kong L, Heng T, Khoeun S, Tum S, Selleck PW, et al. Risk mapping using serologic surveillance for selected One Health and transboundary diseases in Cambodian goats. PLoS Negl Trop Dis. 2023;17:e0011244. 10.1371/journal.pntd.0011244. 37011099 10.1371/journal.pntd.0011244 PMC10101637

[113] Troupin C, Ellis I, Doukouré B, Camara A, Keita M, Kagbadouno M, et al. Seroprevalence of brucellosis, Q fever and Rift Valley fever in domestic ruminants in Guinea in 2017–2019. BMC Vet Res. 2022;18:64. 10.1186/s12917-022-03159-x. 35120506 10.1186/s12917-022-03159-x PMC8815129

[114] Zahid MU, Hussain MH, Saqib M, Neubauer H, Abbas G, Khan I, et al. Seroprevalence of Q Fever (Coxiellosis) in Small Ruminants of Two Districts in Punjab, Pakistan. Vector Borne Zoonotic Dis Larchmt N. 2016;16:449–54. 10.1089/vbz.2015.1852. 10.1089/vbz.2015.1852 27172109

[115] Roest HIJ, Dinkla A, Koets AP, Post J, van Keulen L. Experimental Coxiella burnetii infection in non-pregnant goats and the effect of breeding. Vet Res. 2020;51:74. 10.1186/s13567-020-00797-7. 32471481 10.1186/s13567-020-00797-7 PMC7257221

[116] Sobotta K, Bonkowski K, Liebler-Tenorio E, Germon P, Rainard P, Hambruch N, et al. Permissiveness of bovine epithelial cells from lung, intestine, placenta and udder for infection with Coxiella burnetii. Vet Res. 2017;48:23. 10.1186/s13567-017-0430-9. 28403908 10.1186/s13567-017-0430-9 PMC5389005

[117] Amin F, Ali S, Javid A, Imran M, Rashid MI, Mertens-Scholz K, et al. Sero-Epidemiology of Coxiella burnetii Infection in Small Ruminants in the Eastern Region of Punjab, Pakistan. Pathogens. 2022;11:664. 10.3390/pathogens11060664. 35745517 10.3390/pathogens11060664 PMC9231125

[118] Dean AS, Bonfoh B, Kulo AE, Boukaya GA, Amidou M, Hattendorf J, et al. Epidemiology of brucellosis and q Fever in linked human and animal populations in northern Togo. PLoS ONE. 2013;8:e71501. 10.1371/journal.pone.0071501. 23951177 10.1371/journal.pone.0071501 PMC3741174

[119] Ezatkhah M, Alimolaei M, Khalili M, Sharifi H. Seroepidemiological study of Q fever in small ruminants from Southeast Iran. J Infect Public Health. 2015;8:170–6. 10.1016/j.jiph.2014.08.009. 25270385 10.1016/j.jiph.2014.08.009

