# Supplementary material for: Seroprevalence of Q fever among humans and animals in East Africa: a systematic review and meta-analysis
Source: One Health Outlook. 2026 Apr 3;8:28. doi: 10.1186/s42522-026-00209-4 (PMC13173964; doi:10.1186/s42522-026-00209-4)
Supplement: Supplementary file 1 — Supplementary Material 1 [file 42522_2026_209_MOESM1_ESM.docx]

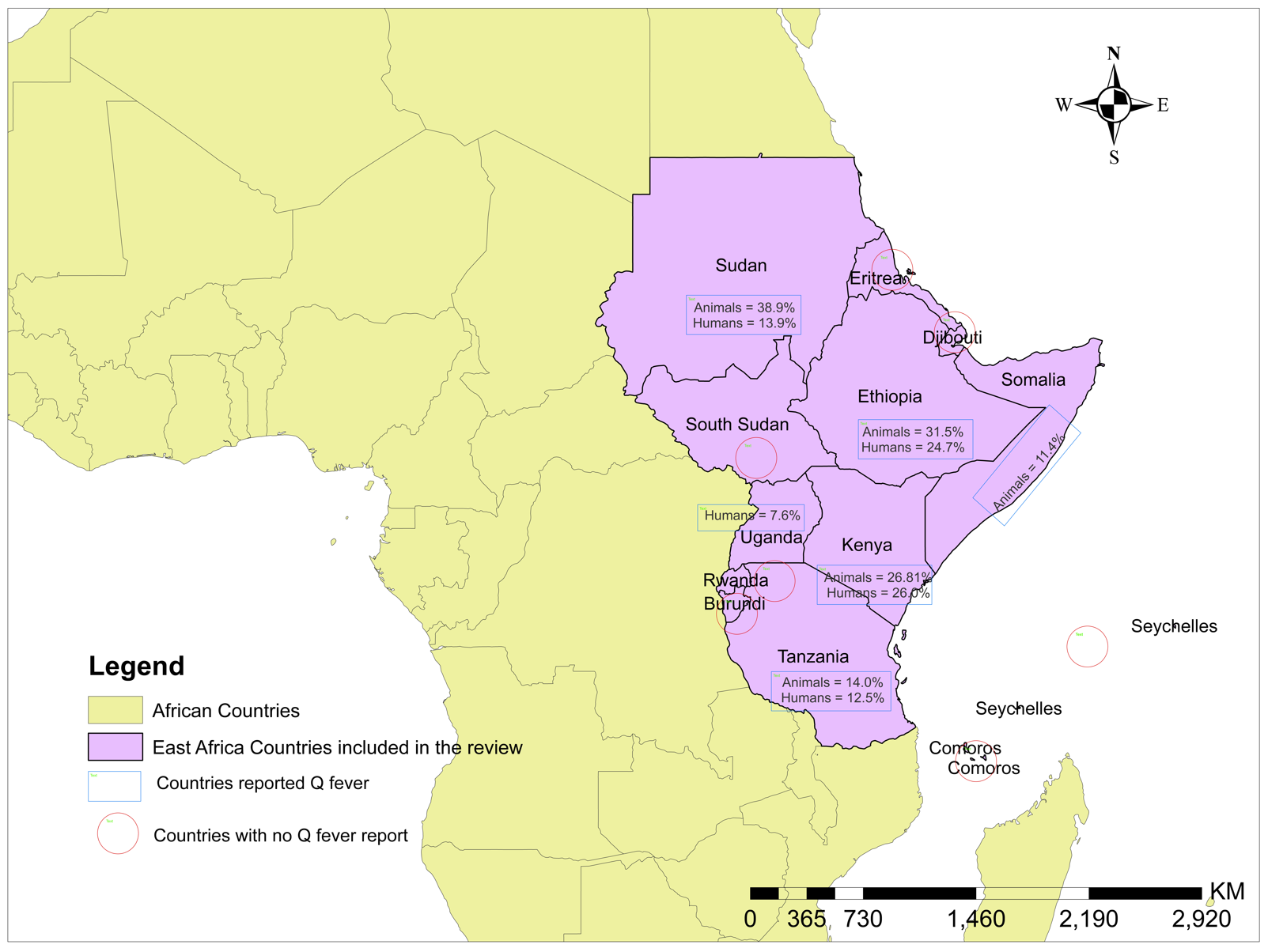


**Fig. S1**. The countries of East Africa with and without Q fever reports


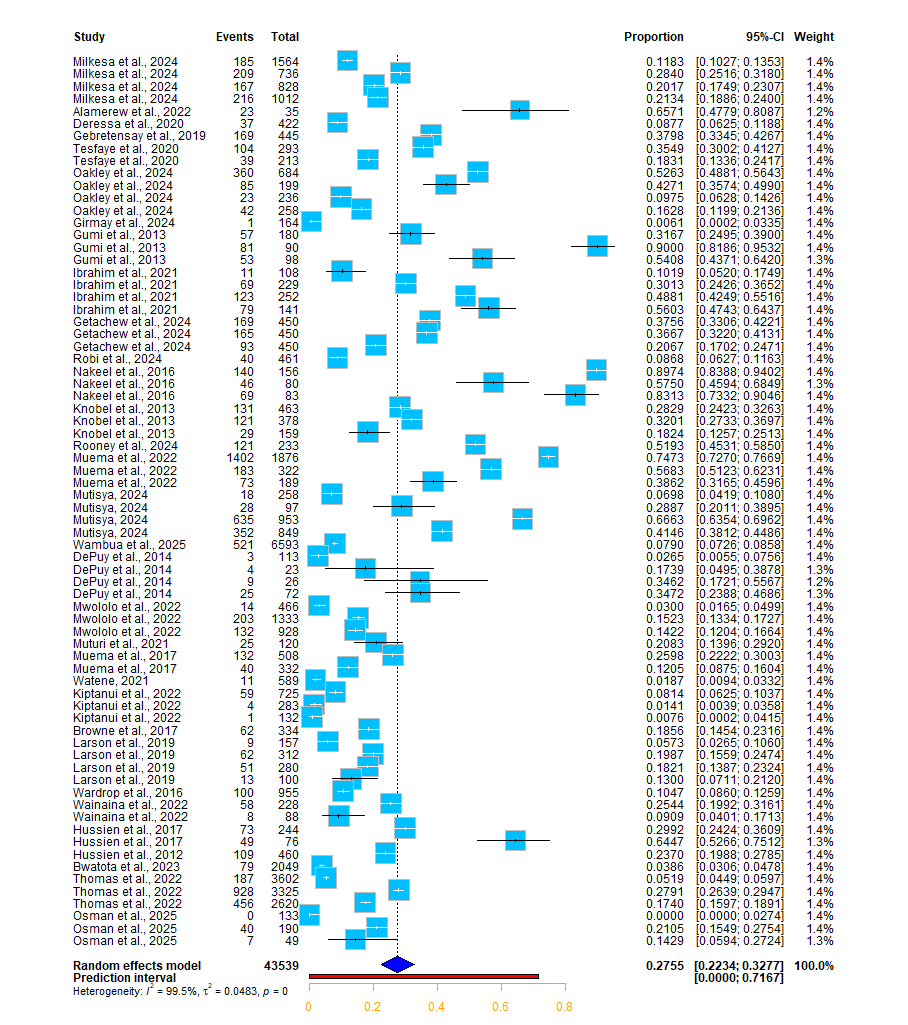


**Fig. S2.** Forest plot showing Q fever seroprevalence in animals


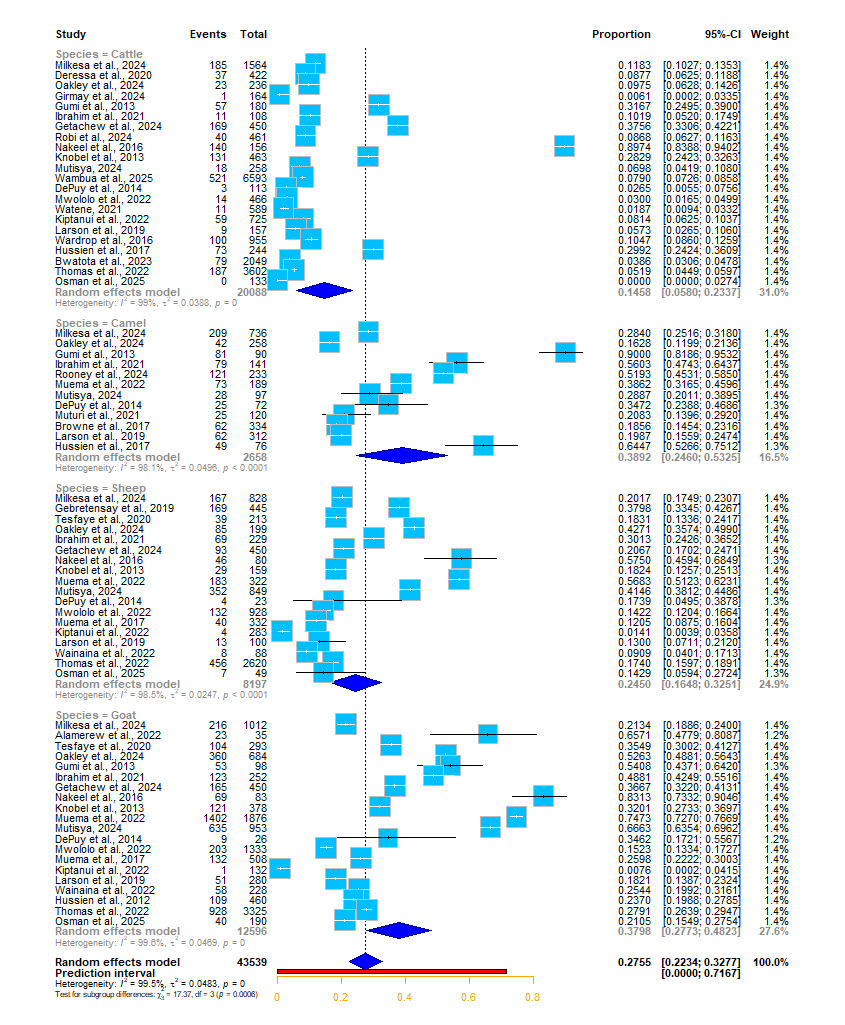


**Fig. S3.** Forest plot showing subgroup analysis of Q fever seroprevalence by animal species


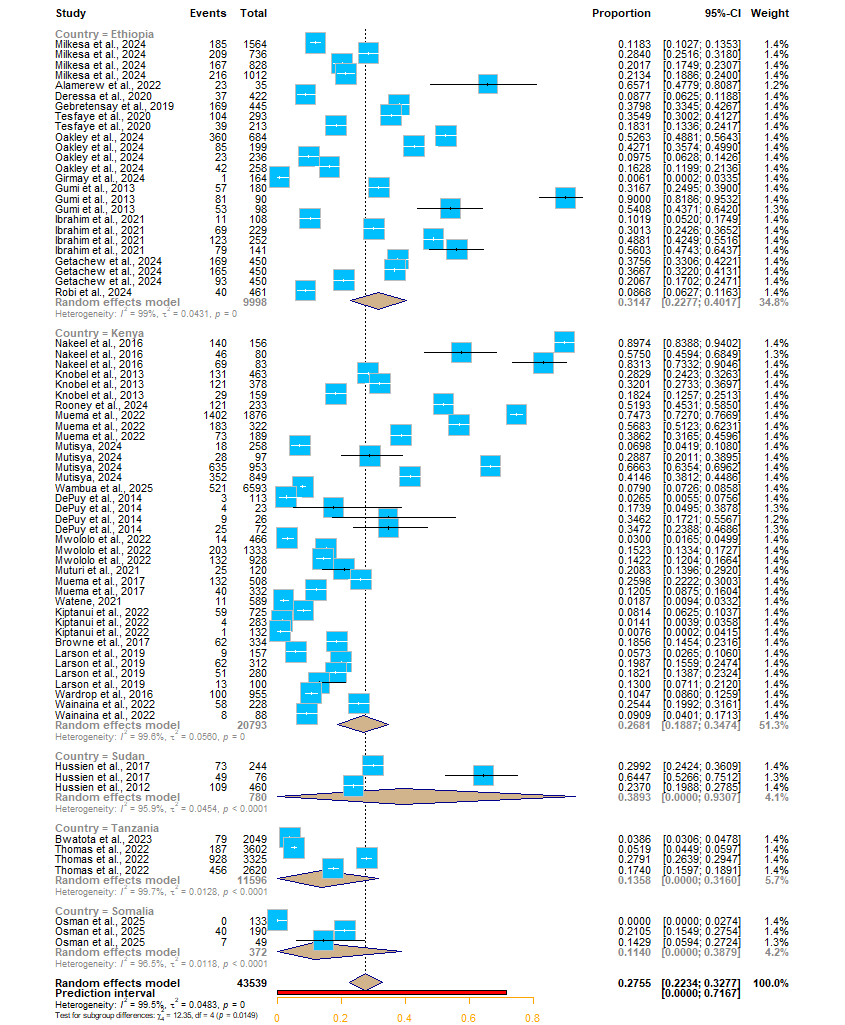


**Fig. S4.** Forest plot showing subgroup analysis of Q fever seroprevalence in animals by country.


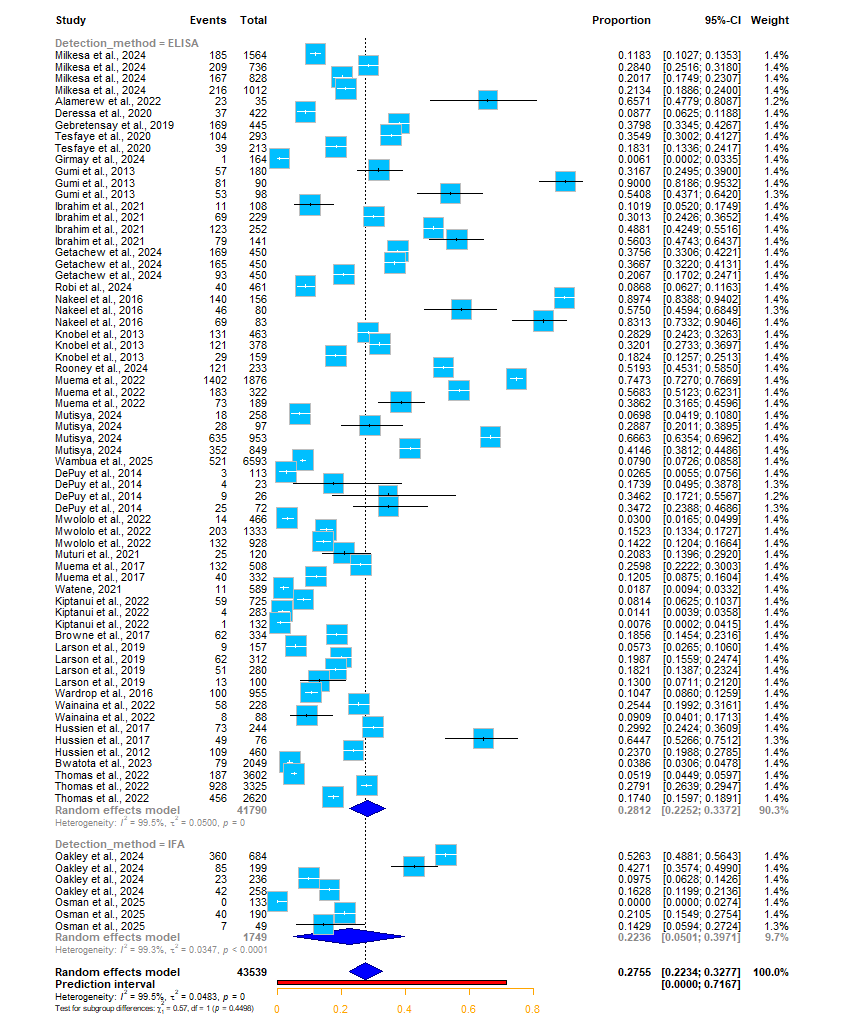


**Fig. S5.** Forest plot showing subgroup analysis of Q fever seroprevalence in animals by detection method.


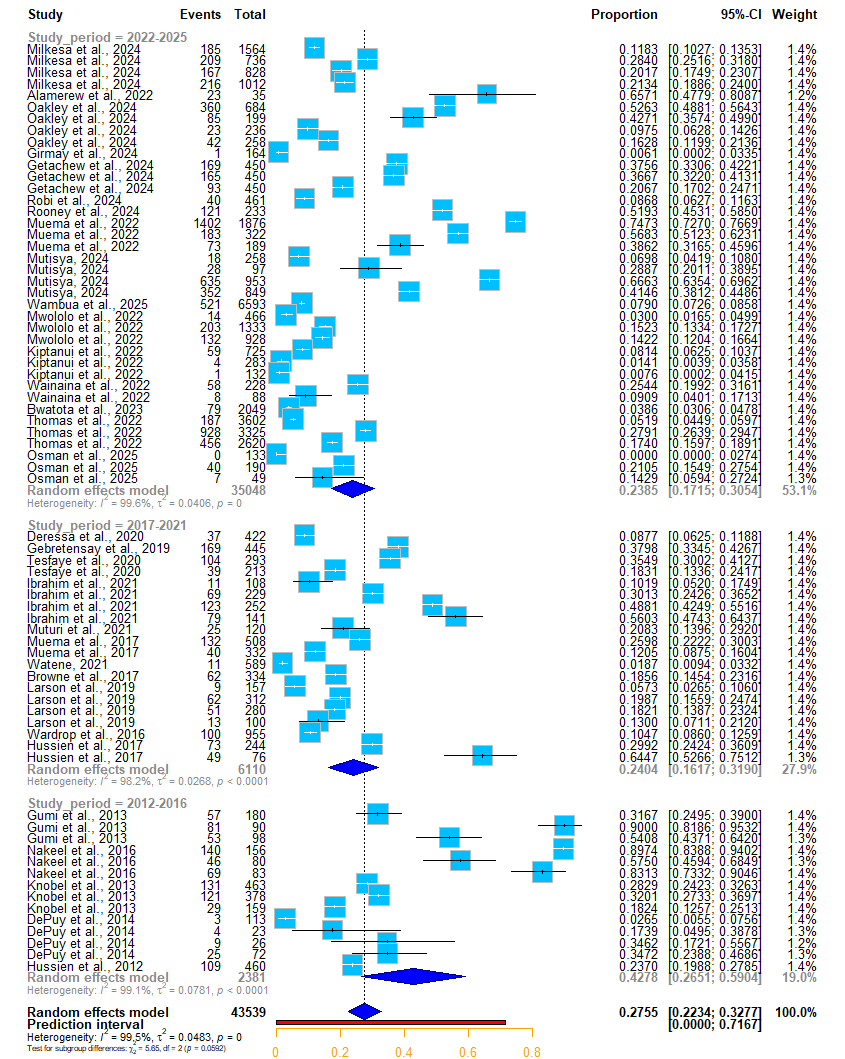


**Fig. S6.** Forest plot showing subgroup analysis of Q fever seroprevalence in animals by study period


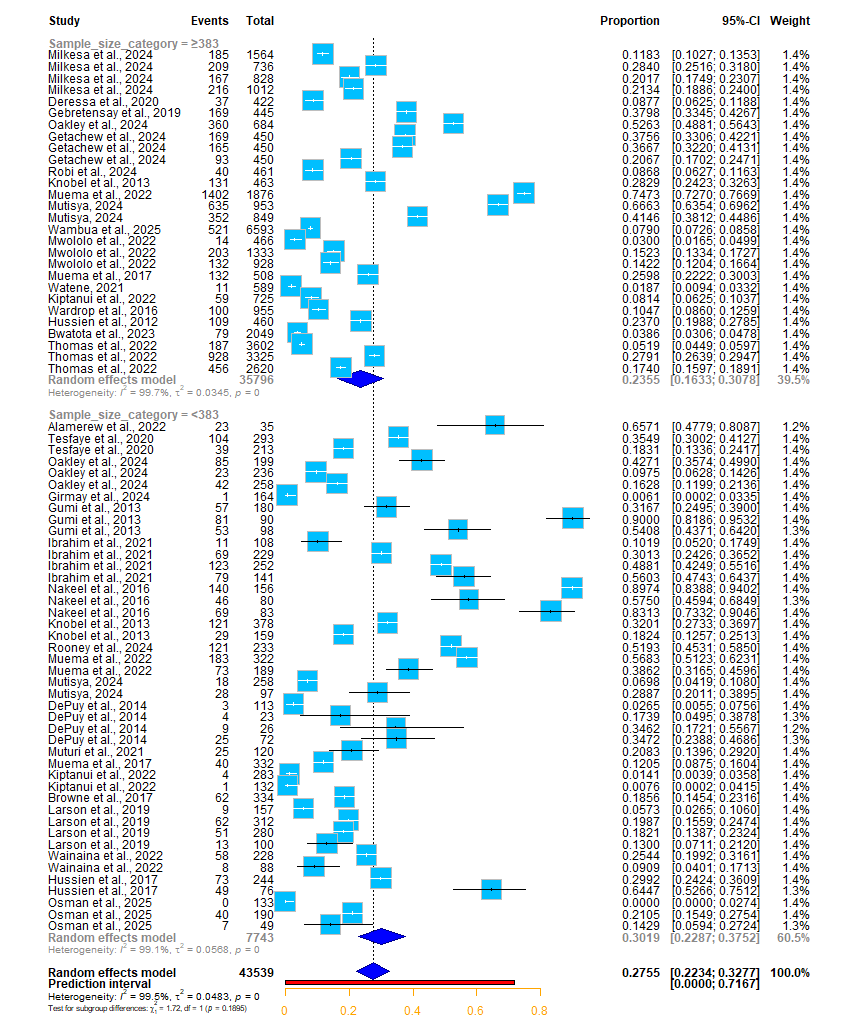


**Fig. S7.** Forest plot showing subgroup analysis of Q fever seroprevalence in animals by sample size category


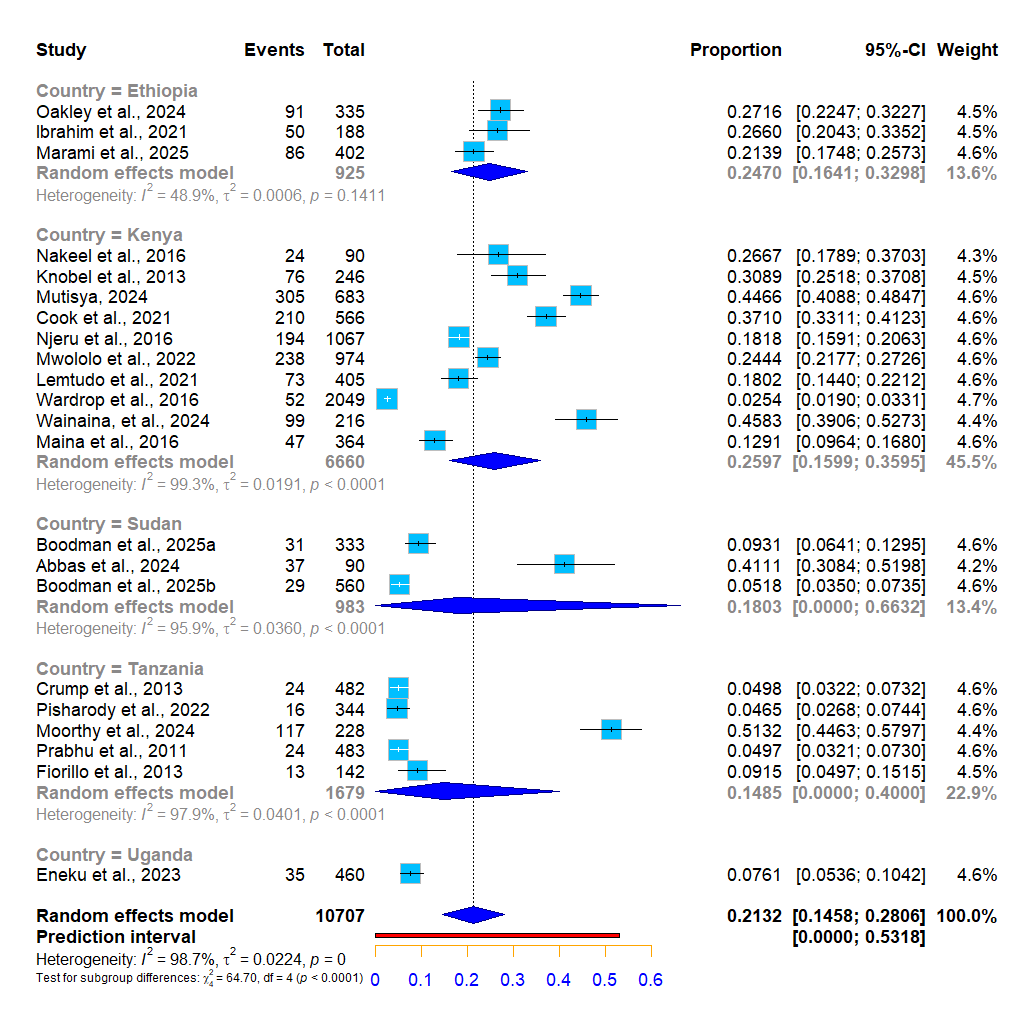


**Fig. S8.** Forest plot showing Q fever seroprevalence in humans.

**
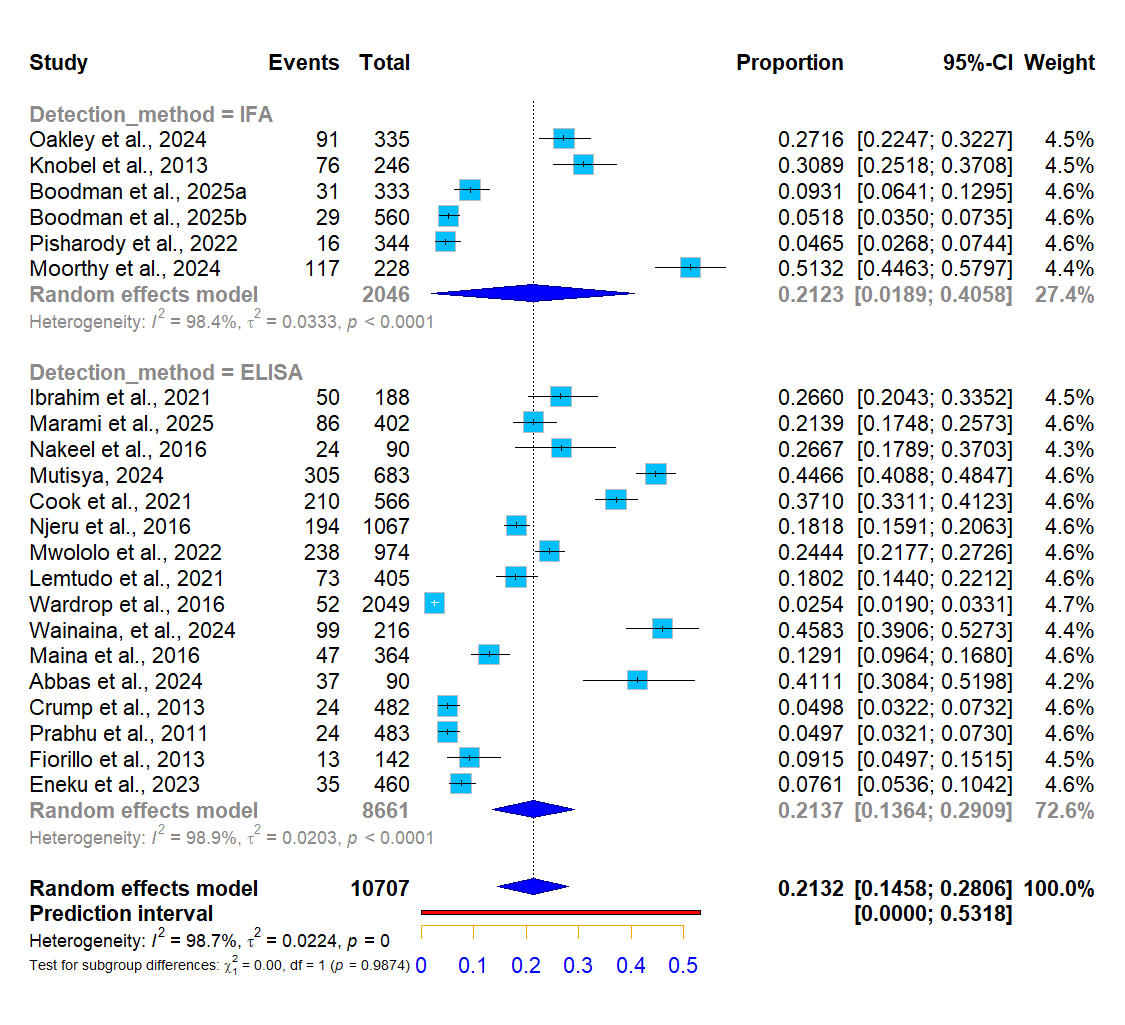
**

**Fig. S9.** Forest plot showing subgroup Q fever seroprevalence in humans by detection methods.

**
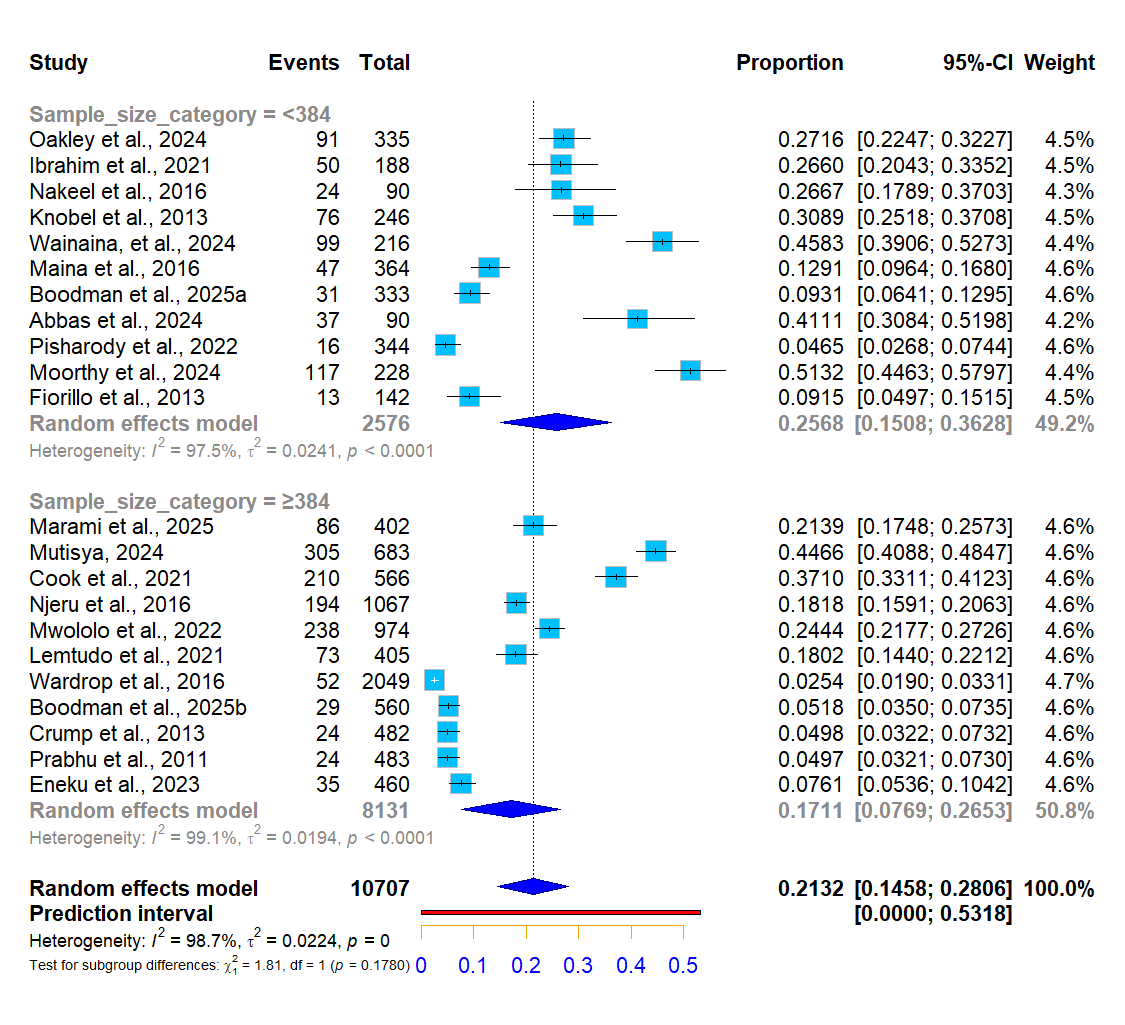
**

**Fig. S10.** Forest plot showing subgroup Q fever seroprevalence in humans by sample size category.

**
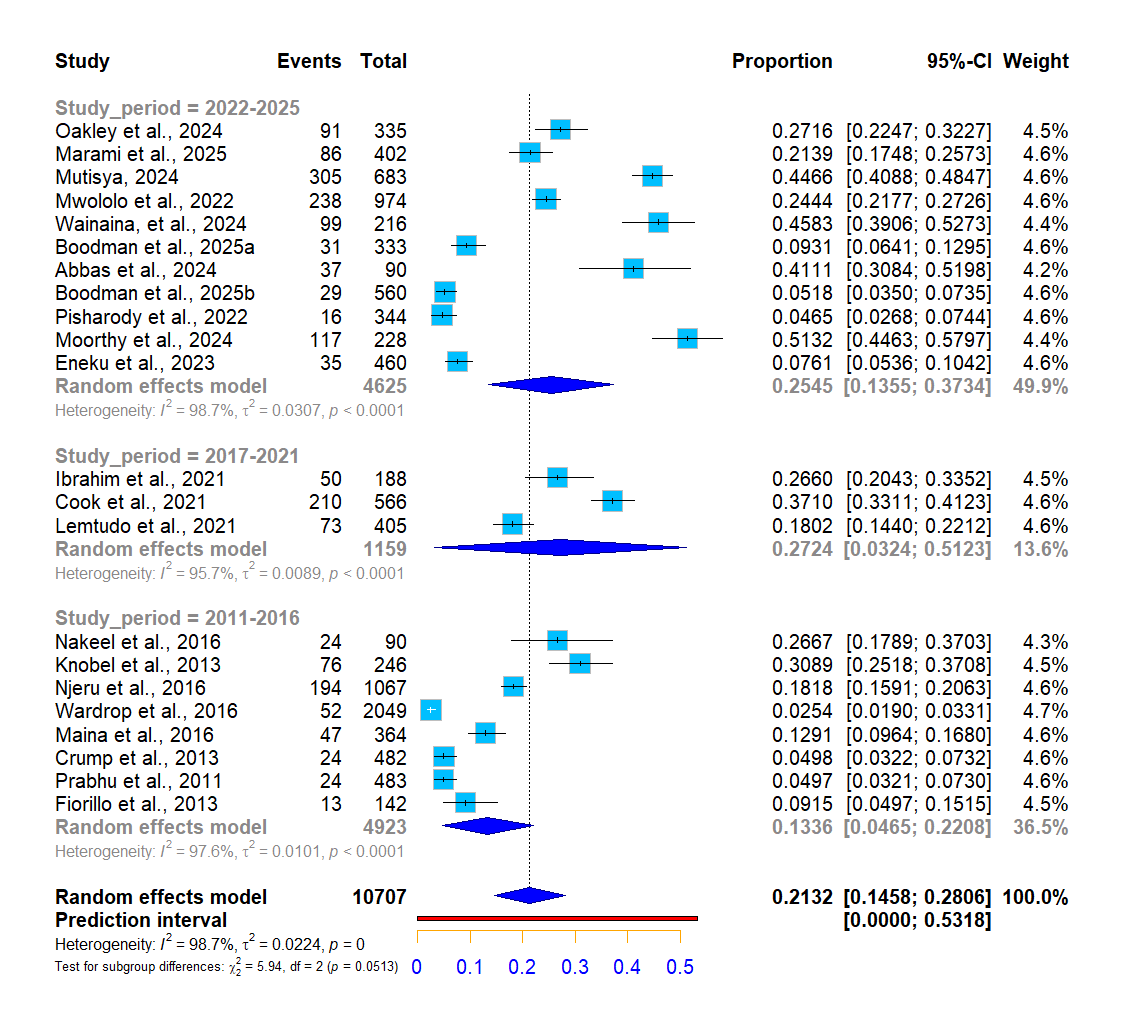
**

**Fig. S11.** Forest plot of Q fever seroprevalence in humans with subgroup study period.


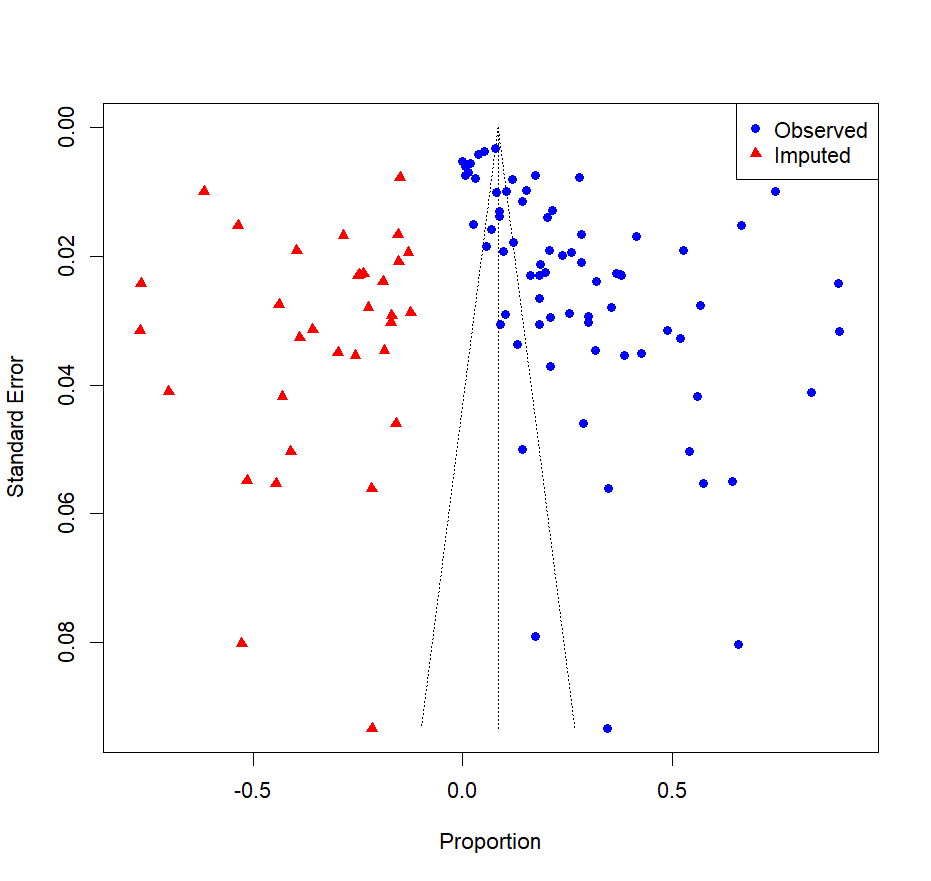


**Fig. S12.** Trim and fill analysis (Duval and Tweedie’s method) of Q fever seroprevalence studies in animals.


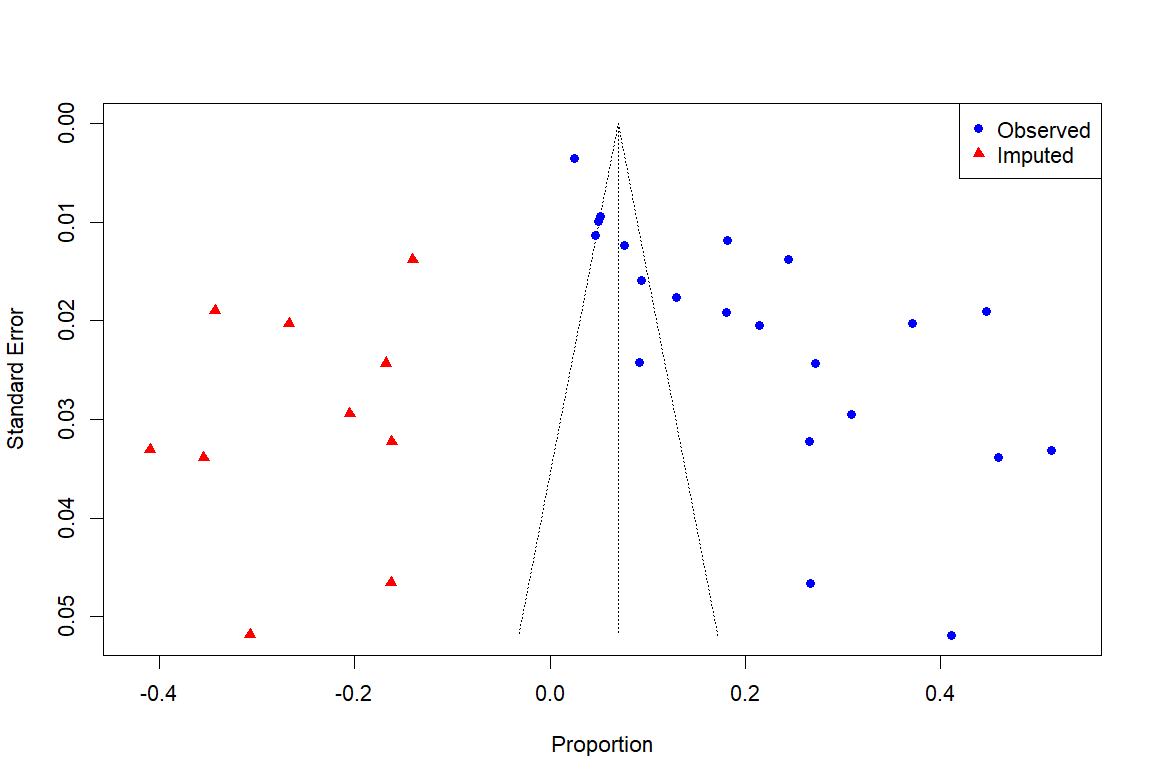


**Fig. S13.** Trim and fill analysis (Duval and Tweedie’s method) of Q fever seroprevalence studies in humans.


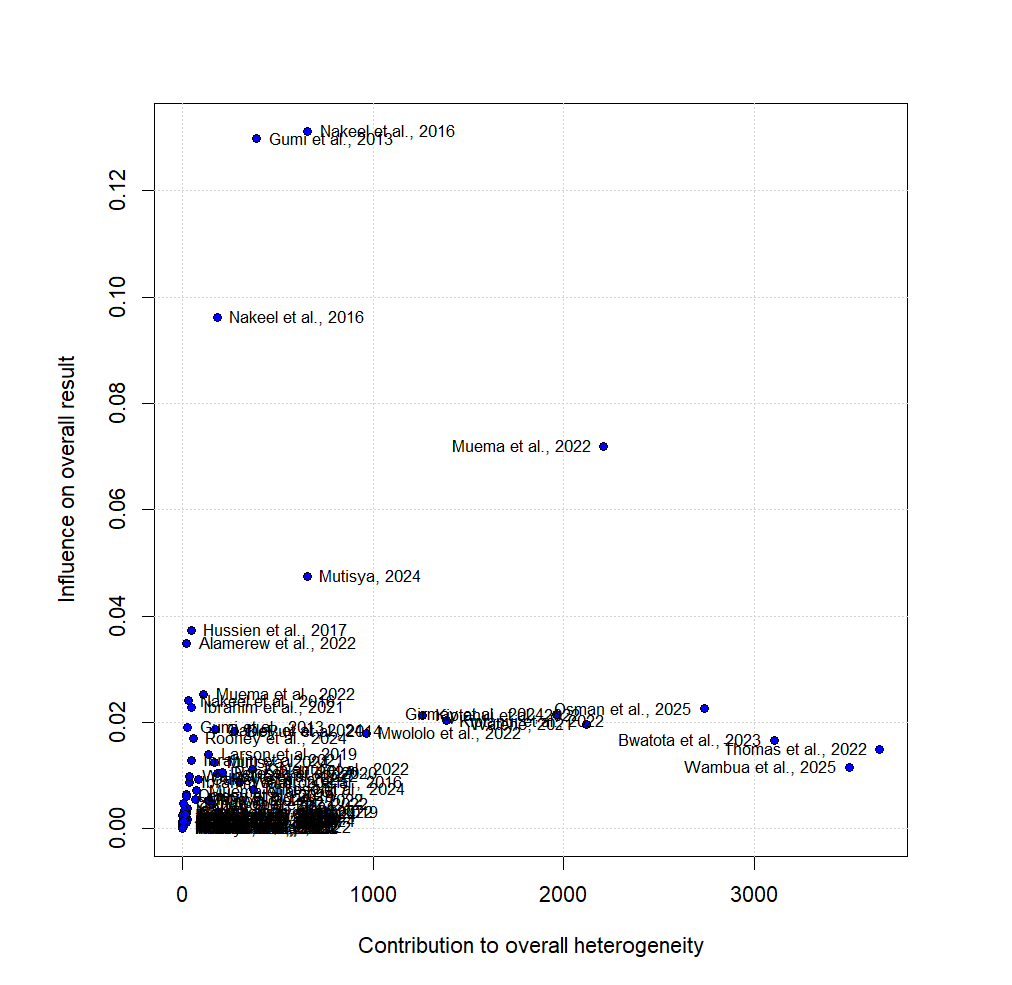


**Fig. S14.** Baujat diagnostic plot showing influential studies to the overall Q fever seroprevalence and heterogeneity in animals.


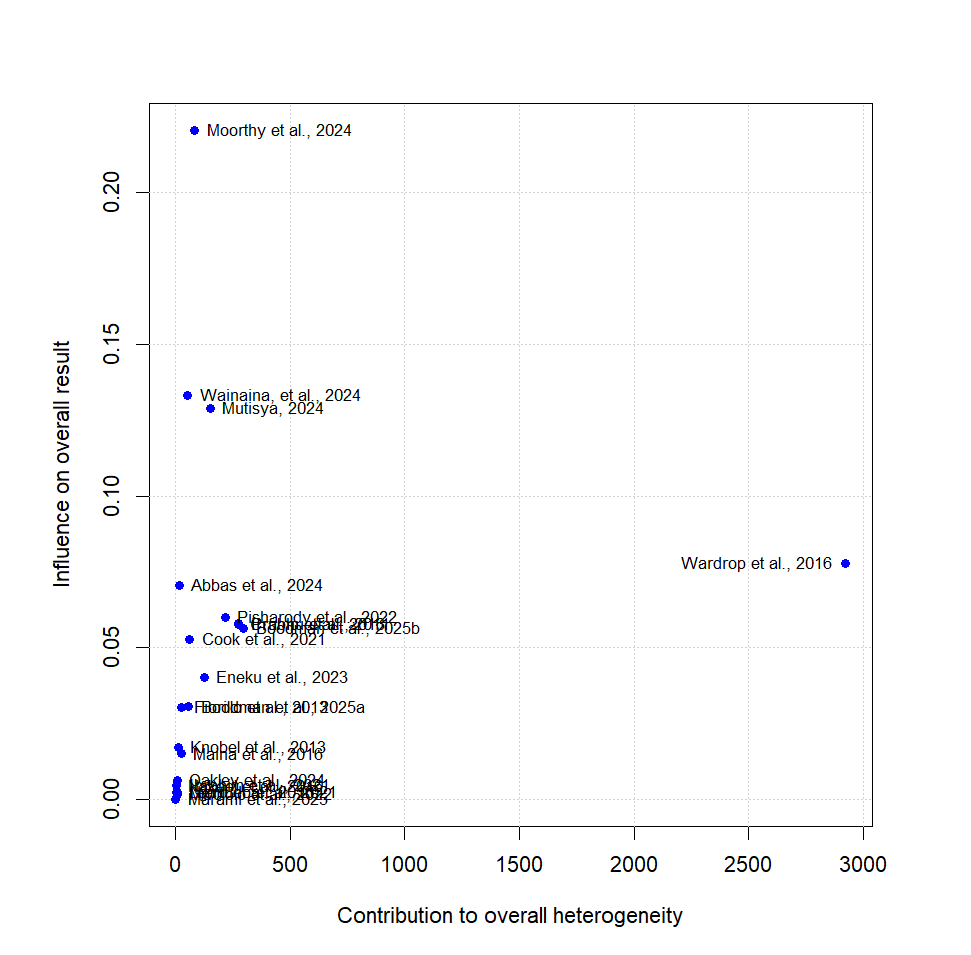


**Fig. S15.** Baujat diagnostic plot showing influential studies to the overall Q fever seroprevalence and heterogeneity in humans.
