# Supplementary material for: Seroprevalence of Q fever among humans and animals in East Africa: a systematic review and meta-analysis
Source: One Health Outlook. 2026 Apr 3;8:28. doi: 10.1186/s42522-026-00209-4 (PMC13173964; doi:10.1186/s42522-026-00209-4)
Supplement: Supplementary file 2 — Supplementary Material 2 [file 42522_2026_209_MOESM2_ESM.docx]

**Table S1**. Included studies in animals and quality assessment

| Author | Was the sample frame appropriate to address the target population | Were study participants sampled in an appropriate way? | Was the sample size adequate? | Were the study subjects and the setting described in detail? | Was the data analysis conducted with sufficient coverage of the identified sample? | Were valid methods used for the identification of the condition? | Was the condition measured in a standard, reliable way for all participants? | Was there appropriate statistical analysis? | Was the response rate adequate, and if not, was  the low response rate managed appropriately? |
| --- | --- | --- | --- | --- | --- | --- | --- | --- | --- |
| Milkesa et al., 2024 | Yes | Yes | Yes | Yes | Yes | Yes | Yes | Yes | Yes |
| Alamerew et al., 2022 | No | No | No | Yes | Yes | Yes | Yes | No | No |
| Deressa et al., 2020 | Yes | Yes | Yes | Yes | Yes | Yes | Yes | Yes | Yes |
| Gebretensay et al., 2019 | Yes | Yes | Yes | Yes | No | Yes | Yes | Yes | No |
| Tesfaye et al., 2020 | Yes | Yes | Yes | Yes | Yes | Yes | Yes | Yes | Yes |
| Oakley et al., 2024 | Yes | Yes | Yes | Yes | Yes | Yes | Yes | Yes | Yes |
| Girmay et al., 2024 | No | No | Yes | Yes | Yes | Yes | Yes | Yes | Yes |
| Gumi et al., 2013 | Yes | Yes | No | Yes | Yes | Yes | Yes | No | No |
| Ibrahim et al., 2021 | Yes | Yes | Yes | Yes | Yes | Yes | Yes | Yes | Yes |
| Getachew et al., 2024 | Yes | No | Yes | No | Yes | Yes | Yes | Yes | Yes |
| Robi et al., 2024 | Yes | Yes | Yes | Yes | Yes | Yes | Yes | Yes | Yes |
| Nakeel et al., 2016 | No | Yes | No | No | Yes | Yes | Yes | No | Yes |
| Knobel et al., 2013 | Yes | Yes | Yes | No | Yes | Yes | Yes | Yes | Yes |
| Rooney et al., 2024 | No | No | Yes | Yes | Yes | Yes | Yes | Yes | Yes |
| Muema et al., 2022 | Yes | No | Yes | Yes | Yes | Yes | Yes | Yes | Yes |
| Mutisya, 2024 | Yes | Yes | Yes | Yes | Yes | Yes | Yes | Yes | Yes |
| Wambua et al., 2025 | Yes | Yes | Yes | Yes | Yes | Yes | Yes | Yes | Yes |
| DePuy et al., 2014 | Yes | No | No | Yes | No | Yes | Yes | No | Yes |
| Mwololo et al., 2022 | Yes | Yes | Yes | Yes | Yes | Yes | Yes | Yes | Yes |
| Muturi et al., 2021 | Yes | Yes | Yes | Yes | Yes | Yes | Yes | Yes | Yes |
| Muema et al., 2017 | Yes | Yes | Yes | Yes | Yes | Yes | Yes | Yes | Yes |
| Watene, 2021 | Yes | Yes | Yes | Yes | Yes | Yes | Yes | Yes | Yes |
| Kiptanui et al., 2022 | Yes | Yes | Yes | Yes | Yes | Yes | Yes | Yes | Yes |
| Browne et al., 2017 | Yes | No | Yes | No | Yes | Yes | Yes | Yes | Yes |
| Larson et al., 2019 | No | Yes | Yes | Yes | Yes | Yes | Yes | Yes | No |
| Wardrop et al., 2016 | Yes | Yes | Yes | Yes | Yes | Yes | Yes | Yes | Yes |
| Osman et al., 2025 | No | No | Yes | No | Yes | Yes | Yes | No | Yes |
| Wainaina et al., 2022 | Yes | No | Yes | Yes | Yes | Yes | Yes | Yes | No |
| Hussien et al., 2016 | Yes | Yes | No | No | Yes | Yes | Yes | No | Yes |
| Hussien et al., 2012 | Yes | No | Yes | No | Yes | Yes | Yes | No | Yes |
| Bwatota et al., 2022b | No | Yes | Yes | No | Yes | Yes | Yes | Yes | No |
| Thomas et al., 2022 | Yes | Yes | Yes | Yes | Yes | Yes | Yes | Yes | Yes |

**Table S2**. Included studies in human and quality assessment

| Author | Was the sample frame appropriate to address the target population | Were study participants sampled in an appropriate way? | Was the sample size adequate? | Were the study subjects and the setting described in detail? | Was the data analysis conducted with sufficient coverage of the identified sample? | Were valid methods used for the identification of the condition? | Was the condition measured in a standard, reliable way for all participants? | Was there appropriate statistical analysis? | Was the response rate adequate, and if not, was the low response rate managed appropriately? |
| --- | --- | --- | --- | --- | --- | --- | --- | --- | --- |
| Oakley et al., 2024 | Yes | Yes | Yes | Yes | Yes | Yes | Yes | Yes | Yes |
| Ibrahim et al., 2021 | Yes | Yes | Yes | Yes | Yes | Yes | Yes | Yes | Yes |
| Marami et al., 2025 | Yes | Yes | Yes | Yes | Yes | Yes | Yes | Yes | Yes |
| Nakeel et al., 2016 | No | Yes | No | No | Yes | Yes | Yes | No | Yes |
| Knobel et al., 2013 | Yes | Yes | Yes | No | Yes | Yes | Yes | Yes | Yes |
| Mutisya, 2024 | Yes | Yes | Yes | Yes | Yes | Yes | Yes | Yes | Yes |
| Cook et al., 2021 | Yes | Yes | Yes | No | Yes | Yes | Yes | Yes | Yes |
| Njeru et al., 2016a | Yes | Yes | Yes | Yes | Yes | Yes | Yes | Yes | Yes |
| Mwololo et al., 2022 | Yes | Yes | Yes | Yes | Yes | Yes | Yes | Yes | Yes |
| Lemtudo et al., 2021 | Yes | No | No | No | Yes | Yes | Yes | Yes | Yes |
| Wardrop et al., 2016 | Yes | Yes | Yes | Yes | Yes | Yes | Yes | Yes | Yes |
| Wainaina et al., 2024 | Yes | Yes | Yes | Yes | Yes | Yes | Yes | Yes | Yes |
| Maina et al., 2016 | No | No | Yes | No | Yes | Yes | Yes | No | Yes |
| Boodman et al., 2025a | Yes | Yes | Yes | No | Yes | Yes | Yes | Yes | Yes |
| Abbas et al., 2024 | No | No | No | No | Yes | Yes | Yes | Yes | Yes |
| Boodman et al., 2025b | Yes | Yes | Yes | No | Yes | Yes | Yes | No | Yes |
| Crump et al., 2013 | No | No | Yes | Yes | Yes | Yes | Yes | No | Yes |
| Pisharody et al., 2022 | Yes | Yes | Yes | Yes | Yes | Yes | Yes | No | Yes |
| Moorthy et al., 2024 | Yes | No | No | Yes | Yes | Yes | Yes | Yes | Yes |
| Prabhu et al., 2011 | Yes | No | Yes | No | Yes | Yes | Yes | Yes | Yes |
| Fiorillo et al., 2013 | No | No | No | Yes | Yes | Yes | Yes | Yes | Yes |
| Eneku et al., 2023 | Yes | Yes | Yes | Yes | Yes | Yes | Yes | Yes | No |
